# Rational Design, Computational Analysis and Antibacterial Activities of Synthesized Peptide-Based Molecules Targeting Quorum Sensing-Dependent Biofilm Formation in *Pseudomonas aeruginosa*

**DOI:** 10.3390/ph18101572

**Published:** 2025-10-18

**Authors:** Shokhan Jamal Hamid, Twana Mohsin Salih, Tavga Ahmed Aziz

**Affiliations:** 1 Department of Pharmacognosy and Pharmaceutical Chemistry, College of Pharmacy, University of Sulaimani, Sulaymaniyah 46001, Iraq; 2 Department of Pharmacology and Toxicology, College of Pharmacy, University of Sulaimani, Sulaymaniyah 46001, Iraq

**Keywords:** antimicrobial peptides, biofilm inhibition, bacterial resistance, MD simulation, molecular docking, quorum sensing, *P. aeruginosa*

## Abstract

**Background/Objective:** The rise in bacterial resistance necessitates novel therapeutic strategies beyond conventional antibiotics. Antimicrobial peptides represent promising candidates but face challenges such as instability, enzymatic degradation, and host toxicity. To overcome these limitations, conjugation and structural modifications are being explored. This study focuses on designing peptide-based inhibitors of the quorum-sensing (QS) regulator LasR in *Pseudomonas aeruginosa*, a key mediator of biofilm formation and antibiotic resistance. **Methods:** Rationally designed tripeptides and dipeptides conjugated with coumarin-3-carboxylic acid and dihydro-3-amino-2-(3*H*)-furanone were evaluated using molecular docking. The most promising ligand–protein complexes were further analyzed using molecular dynamics (MD) simulations conducted with the CHARMM-GUI and AMBER tools to assess the stability of the ligand–protein complex systems, and the binding affinities were evaluated using Molecular Mechanics–Poisson Boltzmann Surface Area (MM-PBSA) calculations. Pharmacokinetic and toxicity profiles were predicted using ADMETLab 3.0. Selected compounds were synthesized via solid-phase peptide synthesis, structurally confirmed by ^1^H NMR and ESI-MS, and tested for antibacterial and antibiofilm activity against *P. aeruginosa* ATCC 27853. **Results:** Computational analyses identified several promising inhibitors with stronger binding affinities than the native autoinducer OdDHL. Coumarin conjugates **C004** and **C006** showed superior docking scores, while MM-PBSA indicated **P004** and **C004** had the most favorable binding energies. MD simulations confirmed stable ligand–protein complexes. ADMET predictions highlighted **C004** and **C006** as having excellent pharmacokinetic properties. Experimental assays showed moderate antibacterial activity (MIC 512–1024 µg/mL) and strong antibiofilm inhibition, particularly for **C004** (83% inhibition at ½ MIC). **Conclusions:** The study demonstrates that peptide–coumarin conjugates, especially **C004**, are promising tools for disrupting QS and biofilm formation in *P. aeruginosa*. Further optimization and in vivo validation are needed to advance these compounds toward therapeutic application.

## 1. Introduction

Antibacterial drugs have provided critical protection against severe bacterial infections for nearly eight decades. However, the emergence and rapid progression of bacterial resistance pose a substantial threat to the efficacy of existing antibacterial therapies, raising the alarming possibility of a post-antibiotic era [1,2]. Therefore, alternative approaches, including the development of innovative antibacterial agents, are urgently needed [3]. Host Defense Peptides (HDPs), also known as antimicrobial peptides (AMPs), represent a promising alternative to traditional antimicrobial agents. These small peptides have shown remarkable efficacy in combating drug-resistant microbial strains [4,5]. The mechanisms of action of AMPs include bacterial membrane disruption via cationic interactions, interference with intracellular processes, such as bacterial DNA/RNA synthesis or protein synthesis, and, in some cases, modulation of the host immune response [6].

AMPs have generated significant interest among medical researchers due to their promising therapeutic potential in combating antimicrobial resistance. Unlike traditional antibiotics, AMPs are particularly effective because bacteria rarely develop resistance against them, positioning these compounds as critical assets in combating drug-resistant infections [7]. Another key advantage of AMPs is their ability to address biofilm-related infections, which pose a serious threat to human health. Biofilm formation inhibits the efficacy of antibiotics and impairs the ability of the immune system to eliminate infections [8]. AMPs can prevent biofilm formation through various mechanisms, including disrupting established biofilms, inhibiting biofilm adhesion, and downregulating quorum sensing (QS) pathways [9,10].

Despite the promising role of AMPs, they continue to be unsuitable for clinical applications because of issues such as structural instability, enzymatic degradation, and potential toxicity to host cells. Furthermore, physiological and pharmacokinetic factors, including interactions with serum components, salt concentrations, and pH variations, can significantly impact AMP activity in vivo. Moreover, the long peptide sequences and the complicated landscape of their structure–activity relationships make rational design of peptides with targeted biological activity challenging. This difficulty is especially apparent when their antimicrobial efficacy is balanced with their toxicity to human cells. To address these limitations, structural modifications, such as the physical and chemical conjugation of AMPs to other biomolecules have been widely recognized as effective strategies to increase their stability, efficacy, and therapeutic potential [11,12]. Several studies have emphasized the benefits of conjugation approaches in improving AMP performance, reducing toxicity, and increasing bioavailability [13,14,15].

One of the most concerning hospital-acquired infections is caused by the Gram-negative bacterium *Pseudomonas aeruginosa* (*P. aeruginosa*), which is a significant threat, particularly to immunocompromised patients [16]. *P. aeruginosa* is an opportunistic pathogen and can form dense biofilms through QS, a cell-to-cell communication mechanism that regulates group behavior at high cell densities [17]. These biofilm matrices create physical and biochemical barriers that impede drug penetration, often requiring antibiotic concentrations up to 1000 times higher than those needed to eradicate planktonic (free-floating) cells [18]. Consequently, this bacterial characteristic contributes to its resistance to most available antibiotics, leading the World Health Organization (WHO) to classify it as a critical priority 1 pathogen in 2017 [19]. Therefore, disruption of bacterial QS is a promising approach to combat bacterial resistance, particularly compared with traditional antibiotics [20]. QS is regulated by the production of small molecules or peptide signals, whose concentrations increase with cell density. These molecules bind to specific receptors, triggering a coordinated response that enhances bacterial virulence [21]. Hence, disrupting QS signaling through non-native compounds, such as small molecules, peptides, or macromolecules, has emerged as an anti-virulence strategy aimed at reducing bacterial pathogenicity [22].

To disrupt QS in Gram-negative bacteria, particularly in *P. aeruginosa*, it is essential to inhibit the signaling molecule or autoinducer known as *N*-acyl L-homoserine Lactone (AHL) [23]. Typically, the concentration of AHL increases proportionally with bacterial population growth [24], reaching a threshold level that triggers binding to LuxR-type intracellular receptors. This interaction activates the transcription of downstream target genes [23,25,26]. The activation of these genes benefits the bacterial community by promoting biofilm formation, the secretion of proteases and toxins, and the deployment of various motility mechanisms, all of which increase bacterial survival and pathogenicity [27,28].

**Figure 1:**
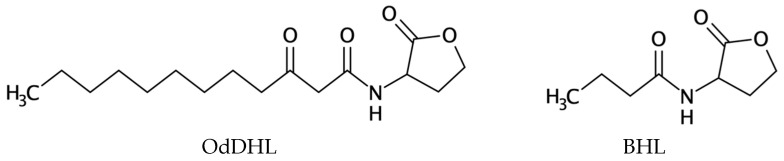
Two-dimensional chemical structures of OdDHL and BHL.

*P. aeruginosa* possesses three LuxR-type receptors: LasR, Rh1R, and QscR. Among these, LasR is the most critical target for inhibitory molecules because of its essential role in regulating QS-related virulence. The activation of LasR drives the expression of key virulence phenotypes, including the production of proteases, pyocyanins, and rhamnolipids, which contribute significantly to pathogenicity [21,29]. As shown in Figure 1, this bacterium synthesizes two primary AHL signaling molecules: *N*-3-oxo-dodecanoyl-L-homoserine lactone (OdDHL, 3O-C12-HSL) and *N*-butyryl-L-homoserine lactone (BHL, C4-HSL). These molecules modulate the expression of virulence factors either directly or indirectly, underscoring their essential role in bacterial communication and pathogenicity [30,31].

**Figure 2:**
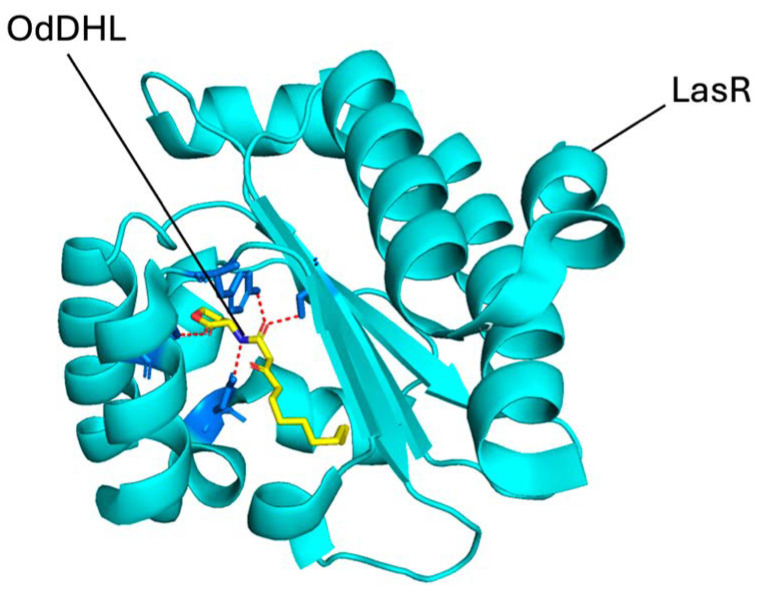
A new cartoon representation of the monomeric LasR–OdDHL complex system. The structural coordinates for this visualization were obtained from the Protein Data Bank (PDB) using the entry code 2UV0.

LasR comprises two distinct, independently folded regions: the larger amino-terminal ligand-binding domain (LBD) and the smaller carboxy-terminal DNA-binding domain (DBD). Binding of its native autoinducer, AHL, to the LBD likely stabilizes the monomeric form of LasR, facilitating the dimerization of two LasR subunits. This ligand-bound homodimer subsequently interacts with DNA, inducing transcriptional changes [21,32]. As depicted in Figure 2, the OdDHL autoinducer is deeply embedded within the ligand-binding pocket of the LasR protein, forming the hydrogen bonds and hydrophobic interactions critical for its function.

This study aimed to identify potential molecules capable of inhibiting the LasR protein, a major regulator in the QS pathway of *P. aeruginosa*. Diverse peptide-based compounds, including tripeptides, dipeptide–coumarin conjugates, and dipeptide–furanone conjugates, have been designed via rational design approaches. These molecules were specifically intended to target the LBD at the N-terminal region of LasR. Molecular docking was employed to predict the binding affinities and analyze the interaction profiles between the ligands and the LasR protein. To further validate in silico results, Molecular Dynamics (MD) simulations were conducted, allowing the assessment of the stability, behavior, and dynamic motions of the ligand-protein complexes over time. Additionally, the pharmacokinetic and toxicity profiles of the selected compounds were evaluated using computational tools to determine their drug-likeness, safety, and bioavailability. These predictions support the suitability of the identified molecules for further development as novel therapeutic agents that target QS mechanisms. After that, antibacterial activities were achieved for the selected molecules based on the in silico results to validate antibacterial and antibiofilm activity of the novel molecules.

## 2. Results and Discussion

*P. aeruginosa* is an opportunistic pathogen known to cause infections in individuals with underlying conditions such as chronic obstructive pulmonary disease (COPD), cancer, burn injuries, cystic fibrosis, immunodeficiency, and those requiring mechanical ventilation due to severe infections [33]. The biofilms formed by this pathogen on host tissues and medical device surfaces represent a significant barrier to effective antimicrobial treatment. Additionally, *P. aeruginosa* often interacts with various microorganisms in host tissues or organs, exhibiting either pathogenic or non-pathogenic behavior [34]. In this study, 30 peptide-based molecules were designed against the LasR protein as the main regulator of *P. aeruginosa* QS [35]. The anti-QS potential of the compounds was subsequently evaluated in silico for their ability to disrupt signal-mediated biofilm formation. Molecular docking and MD simulations were performed to investigate the capacity of the designed peptide-based ligands to competitively inhibit AHL binding to the LasR protein.

### 2.1. Molecular Docking

A molecular docking study was performed on the designed molecules to identify potential competitive inhibitors of the LasR protein. The results revealed that eleven compounds presented higher negative binding energies than did OdDHL, the natural autoinducer. Among these, six compounds (**C002**, **C004**, **C006**, **F007**, **F008**, and **P004**) surpassed the ΔG of the autoinducer by more than −0.6 kcal/mol. All the designed molecules fell within the small-molecule range (MW < 500 g/mol), consistent with the autoinducer, and were categorized as peptide or peptide conjugate molecules [36]. The coumarin conjugated peptides **C004** and **C006** presented the highest negative binding energies, with ΔG values of −10.35 kcal/mol and −9.73 kcal/mol, respectively. However, the average binding energy values of the furanone-conjugated peptides (−8.51 kcal/mol) were greater than those of the coumarin-conjugated peptides (−7.91 kcal/mol). In contrast, tripeptides generally exhibited lowest binding energies (average ΔG = −6.49 kcal/mol), except for P004, which had a notable ΔG of −9.19 kcal/mol (Table 1).

### 2.2. Binding Interactions Between the Selected Compounds and LasR

Peptide molecules have been extensively studied for their potential to inhibit QS and biofilm formation by downregulating the gene expression of LasR [24,37,38,39]. Similarly, numerous studies have demonstrated that coumarin derivatives [40,41,42] and furanone derivatives [43,44,45] can inhibit QS and exhibit antibiofilm activities against *P. aeruginosa*. Consequently, 30 novel compounds were designed, comprising tripeptides, peptide-coumarin conjugates, and peptide-furanone conjugates, with the aim of competitively inhibiting the LasR protein, disrupting QS pathways, and ultimately exhibiting antibiofilm efficacy against *P. aeruginosa*. The LasR binding pocket is predominantly lined with hydrophobic residues, including A, L, F, W, V, and Y. Additionally, the pocket contains two polar residues, S and T, along with one negatively charged polar residue, D. In contrast, the native autoinducer OdDHL is a highly hydrophobic molecule (ClogP = 2.95) (Table 1) due to its long alkyl chain, enabling it to fit deeply into the receptor pocket and establish robust hydrophobic interactions. Furthermore, the 3-oxo group and the oxygen atom of the γ-lactone ring form hydrogen bonds with the main chain of the aspartate residue and the nitrogen atom in the pyrrole group of tryptophan, respectively.

**Figure 3:**
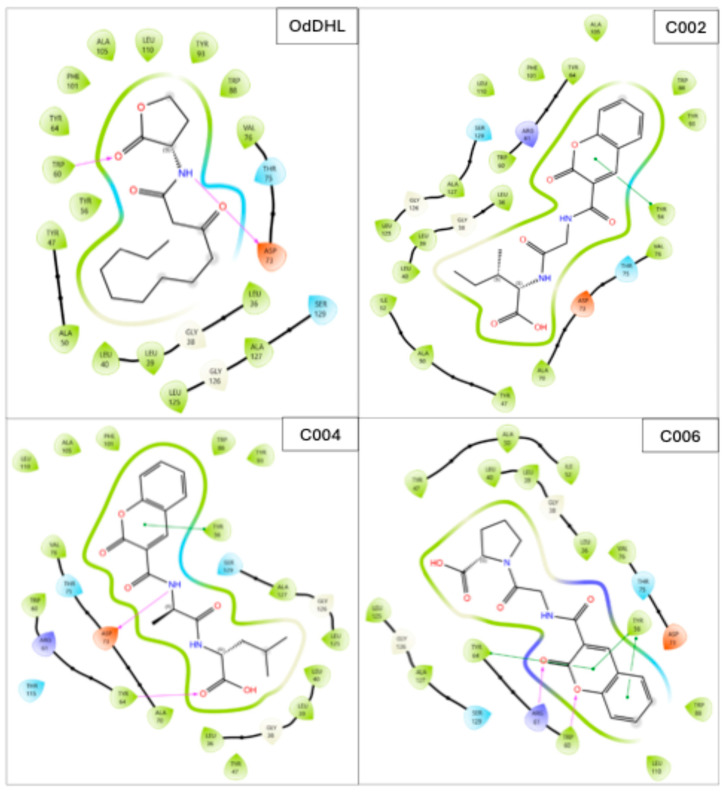

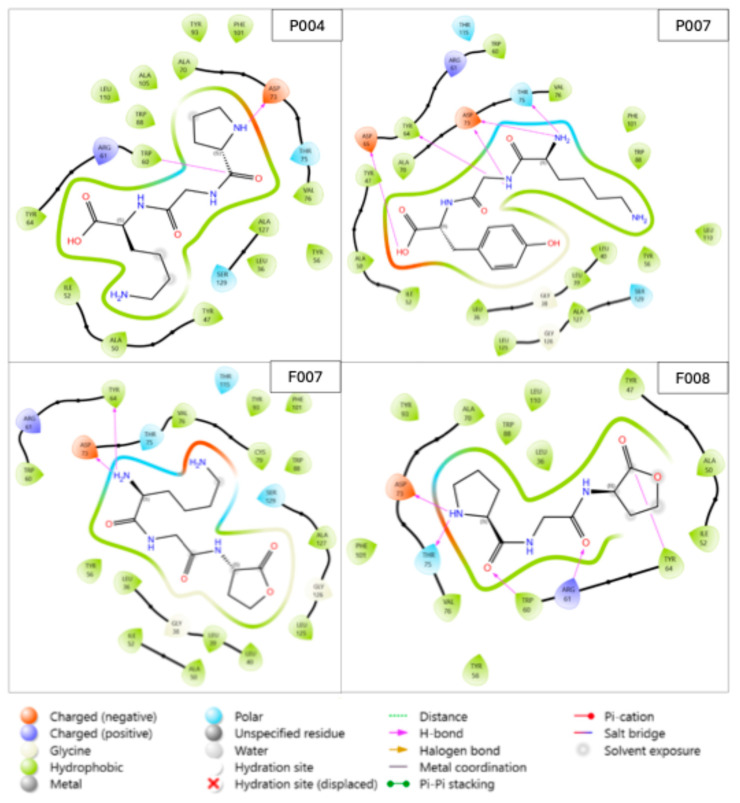
Binding interactions of the selected designed molecules with LasR active site.

The binding interactions between the coumarin-conjugated peptide **C004** and LasR revealed that **C004** had the highest binding affinity among the tested compounds. This can be attributed to its high hydrophobicity (ClogP = 1.53, the highest among the compounds), allowing it to fit optimally into the hydrophobic LasR pocket, facilitated by the isopropyl side chain of the V residue and the benzopyrone ring [46]. Additionally, π–π stacking interactions were observed between the aromatic side chain of the Y56 residue in the protein and the lactone ring of the ligand. Hydrogen bonds also formed between the amino group of the G residue in the ligand and the D73 residue in the protein, as well as between the carboxylic group of the V residue in the ligand and the Y64 residue in the protein (Figure 3).

The compound **C006** demonstrated the second-highest binding affinity, with a slightly less negative ΔG compared to **C004**. This difference is primarily due to the substitution of the V residue with a proline P residue in its structure, which reduces the overall hydrophobicity of **C006** due to the charged amino group present in the pyrrolidine ring. Despite this, **C006** retained two π–π interactions involving tyrosine residues (Y56 and Y64) and the benzopyrone ring of the ligand. Additionally, the oxygen atoms in the α-pyrone ring of the ligand’s coumarin group formed interactions with the W60 and R61 residues within the LasR binding pocket. Compound **F007** exhibited the third-highest binding affinity, primarily due to its increased hydrophilicity. This characteristic facilitated the formation of two hydrogen bonds: one between the main chain amino group of its R residue and the Y64 residue of the LasR binding pocket, and another with the aspartate D73 residue. These hydrophilic interactions replaced hydrophobic contacts and played a significant role in enhancing its binding affinity within the pocket (Figure 3).

### 2.3. MD Simulation Analysis

**Figure 4:**
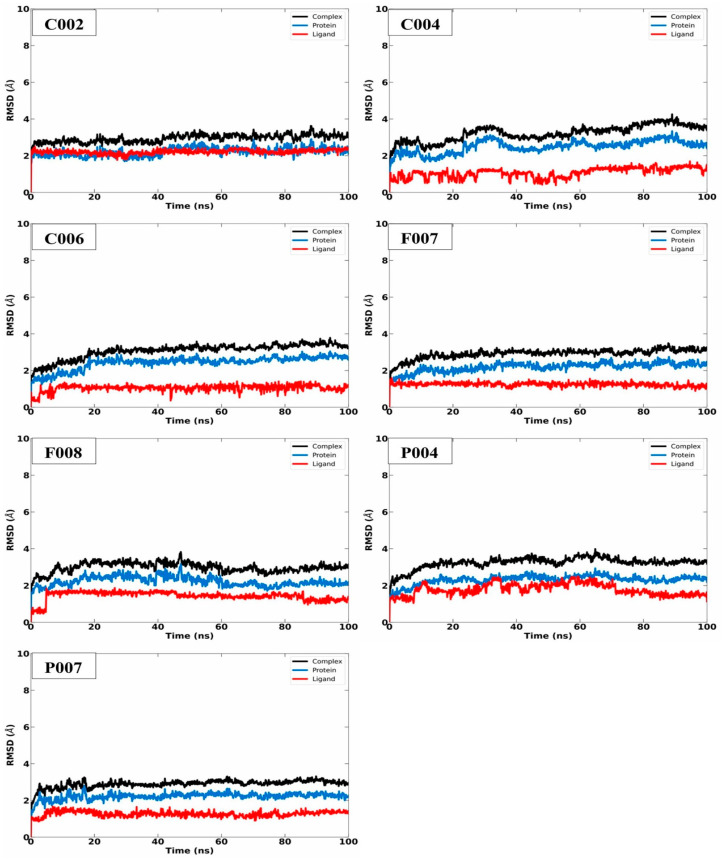
The RMSD of the selected ligand–LasR complex systems.

MD simulations were applied to analyze the interactions, dynamics, and behaviours of the selected ligand-LasR complexes, utilizing trajectory analysis to produce metrics such as RMSD, RMSF, Solvent Accessible Surface Area (SASA), hydrogen bonds (H-bond), center-of-mass (COM) distance, radius of gyration (ROG), and Contact Frequency (CF) analysis. These parameters provided insights into the structural stability, flexibility, solvent interactions, binding strength, and conformational changes in the complexes during the simulation, offering a comprehensive understanding of their functional dynamics [47,48]. One of the most critical metrics for assessing the stability of a ligand-protein complex system is the RMSD, which evaluates the deviation of the protein backbone trajectory over time compared to its initial crystal structure [49]. The results indicated that all complexes reached stability after 2 ns of simulation, with fluctuations remaining below the upper limit of 2 Å—an acceptable range for natural protein motion. Notably, complexes involving **C002**, **F007**, and **P007** exhibited the highest stability, with deviations less than 0.5 Å. The observed fluctuations and overall stability were nearly identical to those of the protein alone, demonstrating minimal influence of ligand conformational changes on the protein structure (Figure 4).

**Figure 5:**
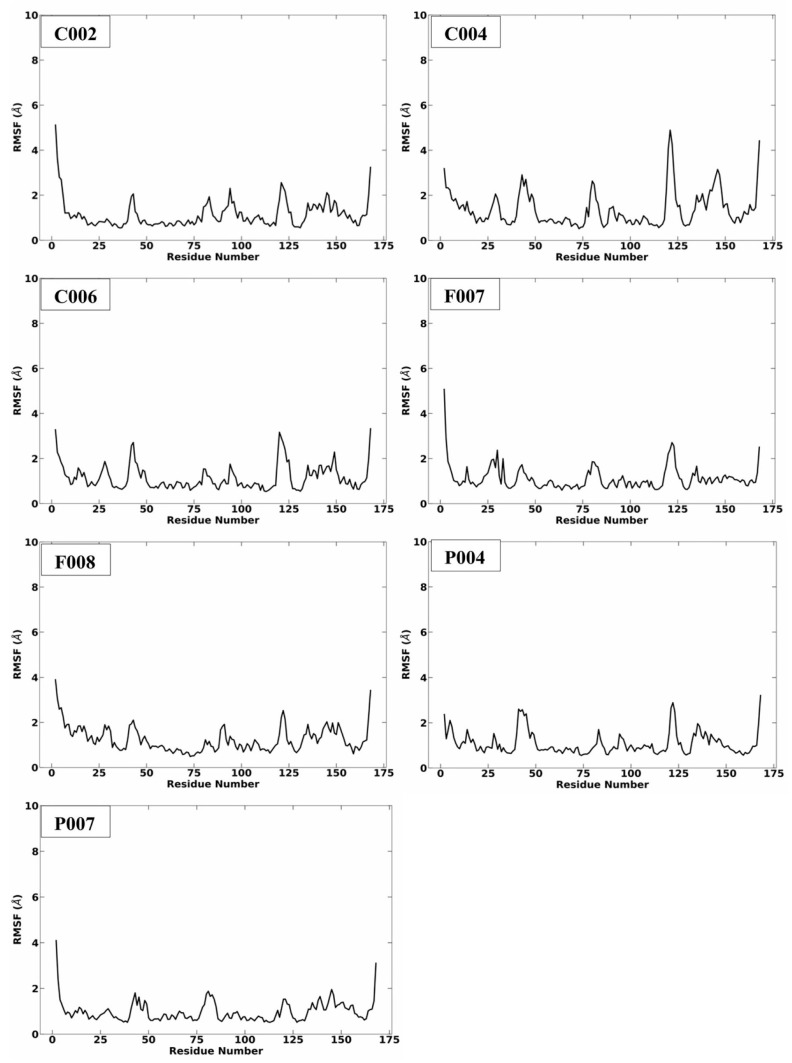
The RMSF of the LasR residues for the ligand–protein complex systems.

Another significant metric in MD simulations is RMSF to measure the flexibility of individual residues within a protein structure over time, providing insights into structural stability and dynamic behaviour. This metric helps identify regions with significant or minimal deviations, such as loops or turns, which tend to exhibit higher fluctuations due to their intrinsic flexibility [50]. In this study, the fluctuation intensity of residues was generally around 1 Å, except for certain residues located within the protein’s loop or turn regions, which demonstrated slightly higher RMSF values (Figure 5).

**Figure 6:**
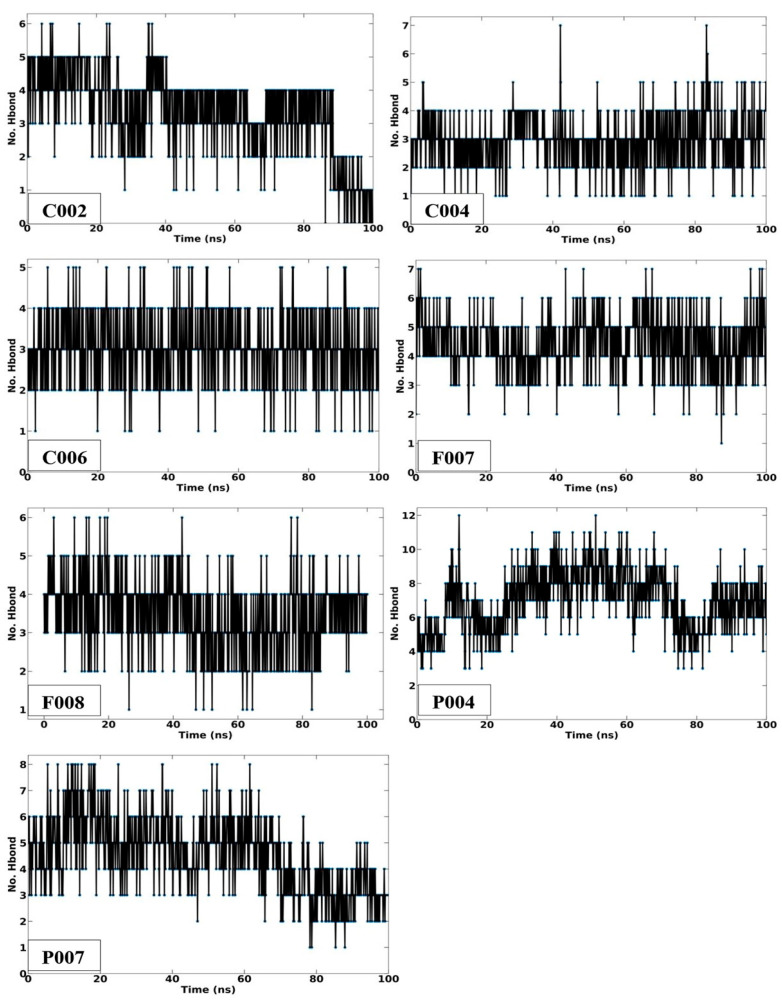
The intermolecular hydrogen bonds between the ligands and LasR protein.

Hydrogen bond analysis involves calculating and visualizing these interactions based on specific geometric criteria, including the angle and distance between the hydrogen, donor, and acceptor atoms. Typically, -OH and -NH groups are considered hydrogen donors, while -O and -N groups act as acceptors by default [51]. The hydrogen bond interactions observed between the designed ligands and the LasR protein during the MD simulations are illustrated in Figure 6. The analysis revealed that the average number of hydrogen bonds formed with the LasR protein was three for compounds **C002**, **C004**, and **C006**. Compounds **F007** and **F008** demonstrated higher levels of interaction, with averages of five and four hydrogen bonds, respectively. In contrast, **P004** lacked stable hydrogen bonds, while **P007** showed an average of four hydrogen bonds during the simulation (Figure 6).

**Figure 7:**
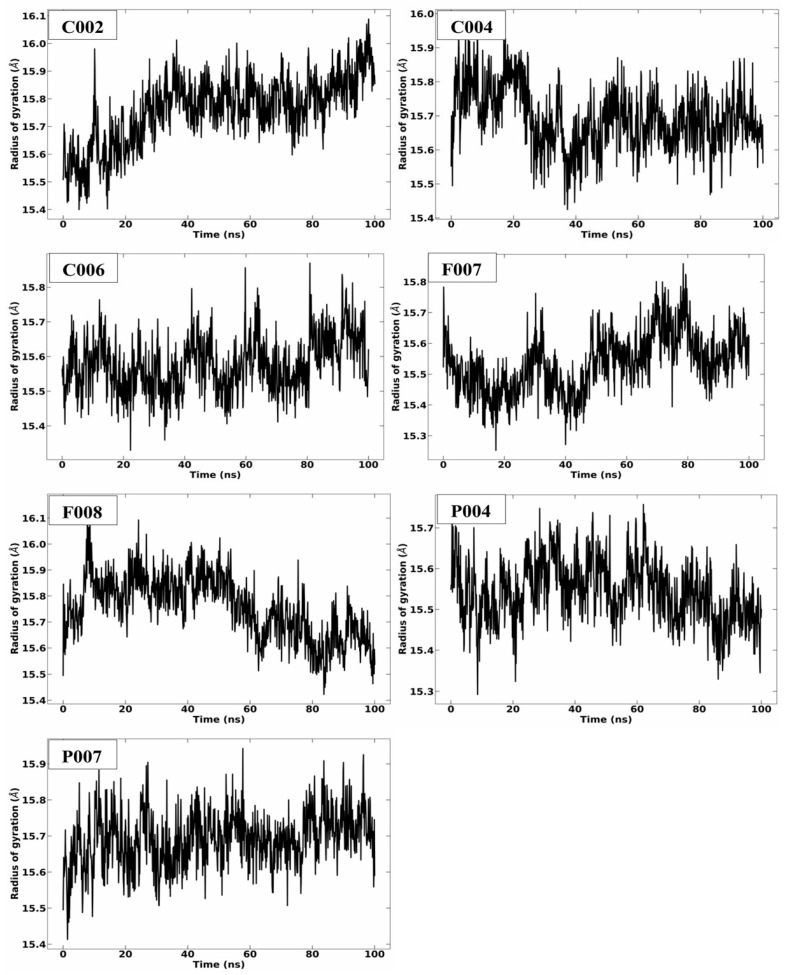
The Rg results of the ligand–LasR complex systems. The protein molecules in all complex systems remained within the normal range of Rg values and demonstrated structural stability throughout the MD simulations.

The Rg quantifies the compactness and folding state of proteins, offering valuable information about the dynamics of macromolecules and the distribution of their mass centers. Typically, larger proteins show higher Rg values [52]. Protein-ligand complexes often display greater Rg values compared to the unbound state. The normal range for Rg lies between 10 and 50 Å. During molecular dynamics simulations, Rg stabilizes into a plateau upon system equilibration; however, significant fluctuations in Rg may indicate conformational changes or protein unfolding [53]. In this study, as depicted in Figure 7, the Rg values for all protein–ligand complexes ranged between 15 and 16 Å, falling within the normal range. This indicates that the protein retained its structural integrity and compaction throughout the simulation. Moreover, the observed Rg values were consistent with those of the co-crystal ligand, suggesting similar compaction and stable dynamics during the simulation (Figure 7).

CF analysis is an MD simulation analysis used to assess the frequency of atomic or residue-level interactions between a ligand and a protein. This approach quantifies the stability and strength of these interactions over time by observing specific distances, often within 3.5–5 Å (for non-covalent interactions), between two atoms based on trajectory files. CF analysis can be implemented to identify key receptor residues crucial for ligand binding and evaluating the overall interaction stability throughout the simulation period [54]. Binding interactions observed in MD simulations are typically considered reliable when their contact frequency exceeds 50%. Interactions with contact frequencies of 80% or higher are particularly significant, as they indicate strong and stable binding between the ligand and protein, which is crucial for maintaining the overall stability of the complex [55].

**Figure 8:**
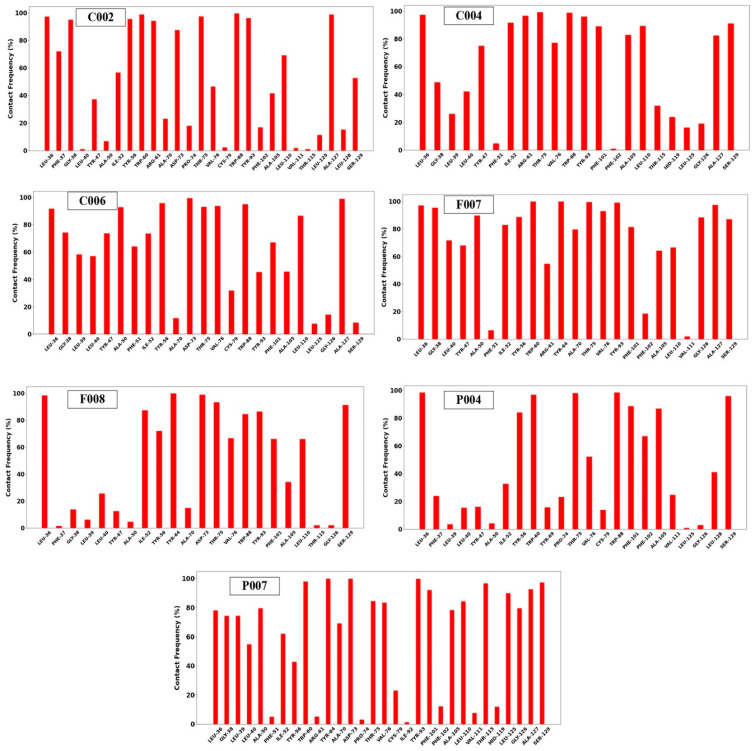
CF analysis of the LasR residues with the selected ligands.

Figure 8 illustrates that each ligand exhibited specific key interactions with the protein residues, with some residues being common across multiple ligands. Compound **C002** demonstrated a CF exceeding 80% with residues L36, G38, Y56, W60, R61, D73, P74, C79, W88, Y93, and L125, and over 50% with F37, I52, L110, and S129 of LasR. Compound **C004** lost high-frequency contacts (>80%) with several residues, including G38, Y56, W60, D73, P74, C79, and L125, but maintained interactions through residues L36, I52, T75, V76, F101, A105, L110, A127, and S129. Notably, residues previously showing a CF > 50% (I52, L110, S129) strengthened their interactions. Additionally, **C004** exhibited a CF > 50% with residues Y47 and G38. Compound **C006** retained a CF > 80% with residues L36, A50, Y56, D73, T75, V76, W88, L110, and A127, and a CF > 50% with G38, L39, L40, Y47, F51, I52, and F101. The **F007** ligand displayed the highest number of interactions, with a CF > 80% for residues L36, G38, A50, I52, Y56, W60, Y64, A70, T75, V76, Y93, F101, G126, A127, and S129, and a CF > 50% for residues L40, Y47, R61, A105, and L110. Ligand **F008** had fewer interactions compared to F007, showing a CF > 80% with L36, I52, Y64, D73, T75, W88, Y93, and S129, and a CF > 50% with Y56, V76, F101, and L110.

Both **C004** and **P004** demonstrated the least interaction, with only eight residues having a CF > 80% and two residues having a CF > 50%. The ligand **P007** had the second-highest number of interactions, with a CF > 80% for residues A50, W60, D73, T75, V76, Y93, F101, A105, L110, T115, L125, G126, A127, and S129, and a CF > 50% for L36, G38, L39, L40, I52, and A70. Notably, all ligands had significant interactions with residues T75 and V76, except for **C002** and L36 was crucial for all the ligands.

**Figure 9:**
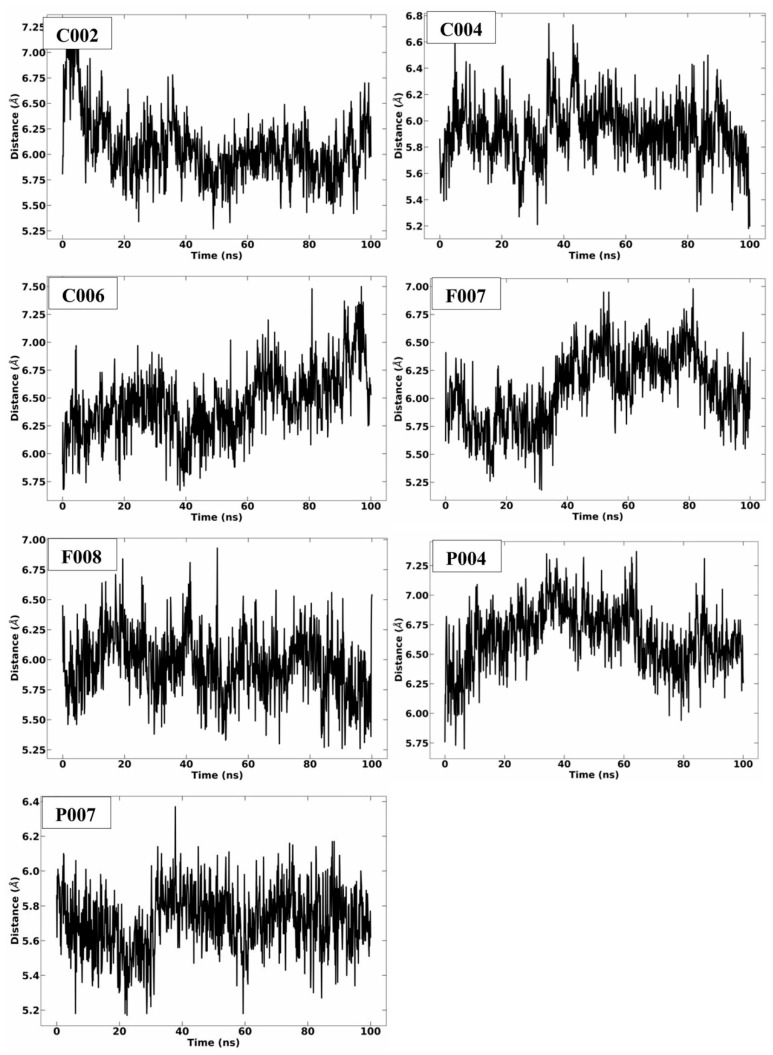
COM distance between LasR protein and the selected ligands, which shows consistent ligand binding with the LasR protein in all the complex systems.

The center of mass (COM) distance, a key parameter in molecular dynamics (MD) simulations, measures the distance between the mass centers of the protein and ligand, providing insights into binding stability and conformational changes. A stable COM distance suggests consistent ligand binding, while fluctuations or increases indicate potential dissociation or movement. Conversely, a decrease may signify ligand rebinding. In this study, protein–ligand interactions were stable, with COM distances between 5 and 7.5 Å because compounds **C006**, **F007**, and **P004** were averaged approximately 6.5 Å; compounds **C002**, **C004**, and **F008** averaged 6 Å, while **P007** averaged 5.8 Å (Figure 9). Combining COM analysis with RMSD and RMSF can enhance understanding of complex dynamics and stability [56,57].

MM-PBSA is a sophisticated method employed to estimate the binding affinity of ligand–protein complexes and to calculate free energy dissociation constants throughout MD simulations [58,59]. The MM-PBSA analysis of the selected ligands revealed that **P004** exhibited the most favourable binding free energy (−55.45 kcal/mol), followed by **C004**, which demonstrated the second-highest negative binding free energy. However, docking results indicated that **C004** exhibited the most negative ΔG overall. The docking results identified **P007** as the ligand with the lowest negative binding energy, whereas the MM-PBSA analysis ranked **P007** as the third-most negative binding energy ligand, following **P004** and **C004** (Table 1 and Table 2).

### 2.4. ADMET Predictions

The early stage of drug discovery is aimed primarily at identifying drug candidates that are both effective and safe. To achieve this, pharmacokinetic properties including absorption, distribution, metabolism, excretion, and toxicity (ADMET) must be thoroughly assessed. These properties are critical for ensuring not only the efficacy but also the safety of the compounds, accompanying the evaluation of their pharmacodynamic characteristics [60]. Over the past four decades, significant advancements in ADMET prediction methodologies have led to the development of various computational platforms that assist researchers in the design and optimization of hit and lead compounds [61]. Among these tools, ADMETlab has emerged as a reliable platform, with its latest version, ADMETlab 3.0, offering improved performance and addressing limitations of earlier versions. This version not only predicts ADMET-related parameters but also includes physicochemical property predictions, providing a comprehensive tool for drug development [62].

The pharmacokinetic properties of the ligands were calculated based on predicted intestinal absorption. Ligands **C002**, **C004**, and **C006** demonstrated excellent intestinal absorption, signifying high bioavailability potential. In contrast, the compound **P004** exhibited poor absorption, which may limit its efficacy. Meanwhile, ligands **F007**, **F008**, and **P007** exhibited moderate absorption levels, indicating potential for optimization in their pharmacokinetic profiles. The distribution properties of the compounds were evaluated by estimating plasma protein binding (PPB) and blood–brain barrier (BBB) permeability, which are crucial for understanding drug bioavailability and central nervous system (CNS) penetration. All ligands exhibited PPB values of ≤90%, indicating a favourable distribution profile with a high proportion of free drug available in the bloodstream. Furthermore, the ligands were predicted to have excellent BBB permeability, with values of <0.1, suggesting their potential to effectively penetrate the CNS. This characteristic enables the compounds to be effective for treating life-threatening CNS infections, including meningitis, encephalitis, and brain abscesses.

Cytochrome (CYP) P450 enzymes play a fundamental role in drug metabolism, with compounds acting either as substrates or inhibitors. Substrates are drugs metabolized by CYP enzymes, influencing their clearance and therapeutic effect. In contrast, inhibitors can block the activity of CYP enzymes, potentially reducing the metabolism of co-administered drugs, leading to enhanced drug effects or toxicity due to drug accumulation.

According to the ADMETlab results, the investigated molecules were generally neither substrates nor inhibitors of CYP enzymes. However, two exceptions were identified: **F007**, which had a low probability of being a CYP2D6 substrate (0.24), and **P007**, which demonstrated a higher probability of being a CYP2D6 substrate (0.87). These findings suggest a limited interaction potential with CYP enzymes for most molecules, except for these specific cases, where metabolic considerations for CYP2D6 may be relevant.

The excretion profiles of the ligands were evaluated using two key parameters: half-life (t_0.5_) and plasma clearance (CL-plasma). As illustrated in Table 3, ligands **C004**, **F007**, **F008**, and **P004** exhibited short half-lives, indicating rapid elimination from the body. Conversely, ligands **C002**, **C006**, and **P007** showed ultra-short half-lives, suggesting even faster clearance rates. In terms of plasma clearance, most ligands demonstrated low clearance, which could be valuable for maintaining therapeutic drug levels over time. However, ligand **P007** displayed moderate clearance, which may result in quicker elimination compared to the other compounds. These findings provide valuable insights into the excretion kinetics of the ligands and their potential impact on pharmacokinetics.

As illustrated in Table 4, the physicochemical properties of the selected ligands were assessed based on molecular weight (MW), topological polar surface area (TPSA), and ClogP. All ligands had MW values below 500 Da, aligning with Lipinski’s rule of five for drug-likeness. However, the TPSA of **P007** exceeded 140 Å^2^, which is predicted to negatively impact its oral bioavailability due to reduced permeability and absorption. Moreover, the ClogP values for **F007**, **F008**, **P004**, and **P007** were below 0, falling outside the optimal range for oral bioavailability. These deviations suggest that these compounds may face challenges in achieving adequate absorption and distribution when administered orally.

The toxicity profiles of the ligands were evaluated based on human hepatotoxicity (H-HT), cardiotoxicity (hERG blockade), and carcinogenicity. All ligands exhibited H-HT values within the range of 0–1, indicating a potential risk of liver damage. Notably, **C002** exposed the lowest H-HT value (0.26), while **C006** had the highest (0.71), emphasizing the need for further hepatotoxicity testing to confirm their safety profiles. For cardiotoxicity, hERG blocker predictions ranged from 0 to 0.19, suggesting a negligible risk of cardiac arrhythmias. Regarding carcinogenicity, none of the ligands, particularly **P004** and **P007**, showed significant carcinogenic potential, with probabilities approaching 0. However, other ligands had carcinogenicity values within 0–1, warranting additional studies to rule out carcinogenic risks conclusively. These findings highlight the necessity of further experimental validation for comprehensive toxicity assessments.

### 2.5. Compounds Chemistry and Characterization

The tripeptides (**P004** and **P007**) and dipeptide–coumarin conjugates (**C002**, **C004**, and **C006**), identified in silico as having high binding affinities, were synthesized via Fmoc-based solid-phase peptide synthesis (SPPS). The products were isolated in moderate to good yields (60–80%). Structural identity and integrity were confirmed by ^1^H NMR and ESI-MS, with spectral data aligning well with the proposed molecular structure, thereby confirming the successful assembly of the target peptides and conjugates. In all cases, the amide NH resonances were observed between δ 9.40 and 8.27. Mass spectrometric analysis revealed ([M + H]^+^) for the tri-peptides, while the coumarin conjugates exhibited ([M]^+^, [M + H]^+^ and [M + 2H]^+^), along with a fragment at *m*/*z* 190 corresponding to the coumarin-3-carboxylic acid core after dipeptide loss, which further substantiating successful conjugation and expected fragmentation pattern (Figures S1–S10).

The use of PyOxim (Oxyma-based phosphonium salt activating reagent) in combination with DIPEA facilitated efficient coupling, minimizing racemization and promoting high-yield amide bond formation. Clean cleavage from Wang resin additionally ensured the purity and structural fidelity of the final compounds [63,64]. Collectively, these results confirm that the designed peptides and peptide-coumarin conjugates are synthetically accessible, structurally robust, and suitable for subsequent evaluation as potential QS inhibitors.

Compound Characterization Data:

- **P004** (PGK)

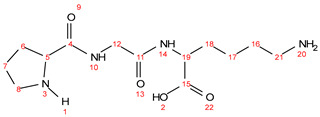


Chemical formula C_13_H_24_N_4_O_4_; calculated molar mass 300.36 g/mol; yield: 60%; off-white crystalline powder; M.P. 198–201 °C; ^1^H NMR (300 MHz, DMSO) δ 12.83 (s, 1H, 2-OH), 8.81 (t, *J* = 5.7 Hz, 1H, 10-NH), 8.30 (d, *J* = 7.8 Hz, 1H, 14-NH), 4.28–4.11 (m, 2H, 19-CH), 3.84 (t, *J* = 5.2 Hz, 2H, 12-CH_2_), ([2.82–2.67 (m, 2H), 2.39–2.18 (m, 1H), 1.88 (ddq, *J* = 11.7, 8.2, 4.8 Hz, 3H), 1.81–1.63 (m, 1H), 1.54 (dq, *J* = 21.8, 8.5 Hz, 3H), 1.34 (q, *J* = 7.9 Hz, 2H)] overlapping protons of proline and lysine CH, CH_2_ and NH). ^13^C NMR (125 MHz, DMSO) δ175.78; C-15, 171.95; C-4, 171.00; C-11, 61.26; C-5, 53.59; C-19, 47.45; C-8, 42.51; C-12, 40.61; C-21, 31.68; C-16, 31.14; C-18, 30.48; C-6, 25.73; C-7, 23.08; C-17. MS (ESI, *m*/*z*): 301 ([M + 1H]^+^).

- **P007** (YGK)

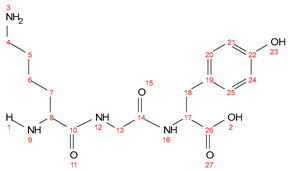


Chemical formula C_17_H_26_N_4_O_5_; calculated molar mass 366.19 g/mol; yield: 68%; white crystalline powder; M.P. 191–193 °C; ^1^H NMR (300 MHz, DMSO) δ 12.84 (s, 1H, 2-OH), 9.31 (s, 1H, 23-OH), 8.67 (t, *J* = 5.6 Hz, 1H, 12-NH), 8.34 (d, *J* = 7.9 Hz, 1H, 16-NH), 7.01 (d, *J* = 7.9 Hz, 2H, 20-H and 25-H), 6.65 (d, *J* = 8.2 Hz, 2H, 21-H and 24-H), 4.40–4.31 (m, 1H, 17-CH), 3.80 (d, *J* = 5.8 Hz, 2H, 13-CH_2_), ([2.74 (q, *J* = 7.4 Hz, 4H), 1.69 (q, *J* = 7.3 Hz, 2H), 1.51 (p, *J* = 7.7 Hz, 2H), 1.35 (q, *J* = 7.7 Hz, 2H), 18, 4, 5, 6 and 7-CH_2_ overlapping]). ^13^C NMR (125 MHz, DMSO) δ172.13; C-26, 170.35; C-14, 168.07; C-10, 156.46; C-22, 130.50; C-20, 130.44; C-25, 129.58; C-19, 115.88; C-24, 115.83; C-21, 54.39; C-17, 54.26; C-8, 43.28; C-13, 40.54; C-4, 37.37; C-18, 34.03; C-7, 29.89; C-5, 23.44; C-6. MS (ESI, *m*/*z*): 367 ([M + 1H]^+^).

- **C002** (IG-coumarin)

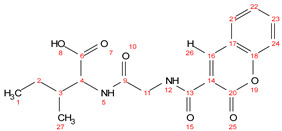


Chemical formula C_18_H_20_N_2_O_6_; calculated molar mass 360.13 g/mol; yield: 80%; white crystalline powder; M.P. 118–120 °C; ^1^H NMR (300 MHz, DMSO) δ 12.68 (s, 1H, 8-OH), 9.08 (t, *J* = 5.2 Hz, 1H, 12-NH), 8.91 (s, 1H, 26-H), 8.27 (d, *J* = 8.4 Hz, 1H, 5-NH), 8.00 (dd, *J* = 7.9, 1.6 Hz, 1H, 21-CH), 7.76 (ddd, *J* = 8.7, 7.3, 1.6 Hz, 1H, 23-CH), 7.56–7.39 (m, 2H, 22 and 24-CH), 4.24 (dd, *J* = 8.4, 5.9 Hz, 1H, 4-CH), 4.07 (d, *J* = 5.2 Hz, 2H, 11-CH_2_), 2.54 (t, *J* = 5.6 Hz, 2H, 2-CH_2_), 1.40 (dtd, *J* = 14.7, 7.4, 4.5 Hz, 1H, 3-CH), 0.91–0.79 (m, 6H, 1 and 27-CH_3_). ^13^C NMR (125 MHz, DMSO) δ175.73; C-6, 169.95; C-9, 166.55; C-13, 160.26; C-20, 153.57; C-18, 144.67; C-16, 132.89; C-23, 129.19; C-21, 125.47; C-22, 118.50; C-17, 116.54; C-24, 115.61; C-14, 57.18; C-4, 42.98; C-11, 36.67; C-3, 25.08; C-2, 15.75; C-27, 11.74; C-1. MS (ESI, *m*/*z*): 360.9 [M^+^], 361 ([M + 1H]^+^), 362 ([M + 2H]^+^).

- **C004** (LA-coumarin)

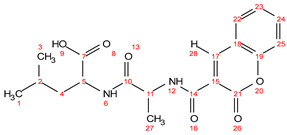


Chemical formula C_19_H_22_N_2_O_6_; calculated molar mass 374.15 g/mol; yield: 76%; white crystalline powder; M.P. 121–124 °C; ^1^H NMR (300 MHz, DMSO) δ 9.40 (d, *J* = 7.2 Hz, 1H, 12-NH), 9.13 (s, 1H, 28-H), 8.64 (d, *J* = 7.9 Hz, 1H, 6-NH), 8.22 (dd, *J* = 7.8, 1.6 Hz, 1H, 22-CH), 7.98 (ddd, *J* = 8.7, 7.3, 1.6 Hz, 1H, 24-CH), 7.79–7.60 (m, 2H, 23 and 25-CH), 4.83 (p, *J* = 6.9 Hz, 1H, 11-CH), 4.48 (td, *J* = 8.4, 6.0 Hz, 1H, 5-CH), 1.95–1.80 (m, 1H, 2-CH), 1.86–1.69 (m, 2H, 4-CH_2_), 1.57 (d, *J* = 6.8 Hz, 3H, 27-CH_3_), 1.10 (dd, *J* = 14.8, 6.3 Hz, 6H, 1 and 3-CH_3_). ^13^C NMR (125 MHz, DMSO) δ174.72; C-7, 172.95; C-10, 166.18; C-14, 160.02; C-21, 153.57; C-19, 146.96; C-17, 132.89; C-24, 129.19; C-22, 125.47; C-23, 118.19; C-18, 116.54; C-25, 115.26; C-15, 52.00; C-5, 49.49; C-11, 41.09; C-4, 24.63; C-2, 22.32; C-1 and 3, 17.39; C-27. MS (ESI, *m*/*z*): 374.9 [M^+^], 375.9 ([M + 1H]^+^), 376.9 ([M + 2H]^+^).

- **C006** (PG-coumarin)

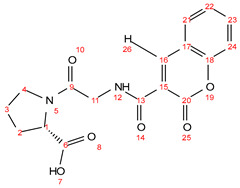


Chemical formula C_17_H_16_N_2_O_6_; calculated molar mass 344.10 g/mol; yield: 71%; off-white crystalline powder; M.P. 168–171 °C; ^1^H NMR (300 MHz, DMSO) δ 13.20 (s, 1H, 7-OH), 9.24 (t, *J* = 4.5 Hz, 1H, 12-NH), 8.96 (s, 1H, 16-H), 8.04 (d, *J* = 7.8, 1.8 Hz, 1H, 21-CH), 7.78–7.69 (m, 2H, 22 and 24-CH), 7.54 (d, *J* = 8.4 Hz, 1H, 23-CH), 4.23 (t, *J* = 4.2 Hz, 2H, 11-CH_2_), 3.64–3.42 (m, 1H, 1-CH), [2.27–2.07 (m, 1H), 2.04–1.82 (m, 3H), 1.32–1.16 (m, 1H), overlapping CH of proline]. ^13^C NMR (125 MHz, DMSO) δ176.29; C-6, 169.64; C-9, 165.68; C-13, 160.46; C-20, 152.19; C-18, 147.97; C-16, 132.89; C-23, 129.19; C-21, 125.47; C-22, 118.50; C-17, 116.59; C-15, 116.54; C-24, 59.19; C-1, 46.23; C-4, 42.73; C-11, 30.15; C-2, 23.78; C-3. MS (ESI, *m*/*z*): 344.8 [M^+^], 345.9 ([M + 1H]^+^), 346.8 ([M + 2H]^+^).

### 2.6. Antibacterial Test of the Synthesized Compounds

#### 2.6.1. Minimum Inhibitory Concentration (MIC)

The synthesized peptide-based compounds revealed moderate antibacterial activity against *P. aeruginosa* ATCC 27853. Dipeptide-coumarin conjugates (**C002**, **C004**, and **C006**) exhibited MIC values of 512 µg/mL, higher than the standard antibiotic azithromycin (256 µg/mL), while tripeptides (**P004** and **P007**) showed higher MIC values of 1024 µg/mL, indicating comparatively lower inhibitory potency (Table 5). The relatively enhanced potency of the coumarin conjugates is likely due to the coumarin moiety, which increases the lipophilicity and facilitates penetration through the Gram-negative outer membrane. These findings are consistent with previous reports on antimicrobial peptides, where bactericidal activity often involves disruption of the bacterial membrane via interactions with lipopolysaccharides [14,15].

Although the MIC values are higher than those of standard antibiotic azithromycin, these findings are consistent with earlier studies on novel peptide conjugates targeting *P. aeruginosa*, where initial scaffolds typically show moderate activity due to an intrinsic resistance mechanism, such as efflux pumps and low outer membrane permeability [38,65]. Overall, the observed activity suggests that these synthesized compounds function as promising tools, and future optimization of hydrophobic/hydrophilic balance, charge distribution, and conjugation strategy may enhance their antibacterial potency.

#### 2.6.2. Antibiofilm Activity Assessment

**Figure 10:**
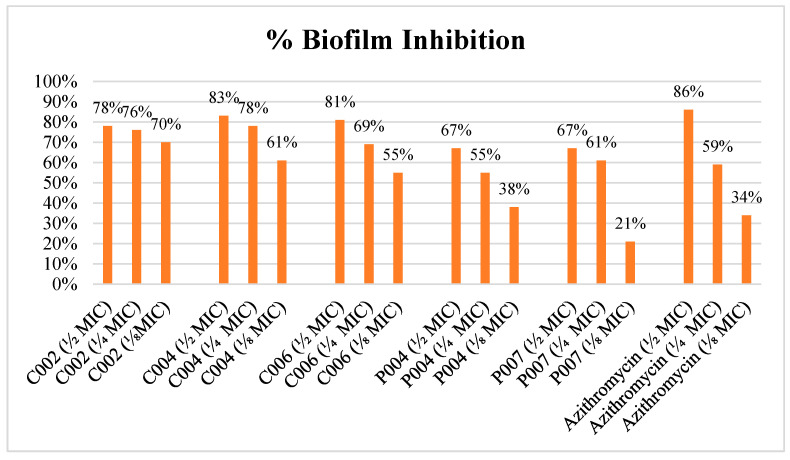
Inhibition of biofilm formation by the peptide-based compounds and azithromycin at sub-inhibitory concentrations (½ MIC, ¼ MIC, and ⅛ MIC).

At sub-MIC levels, the tested compounds demonstrated differential antibiofilm activity (Figure 10). Among the coumarin conjugates (**C002**, **C004**, and **C006**), **C004** exhibited the strongest inhibitory effect, achieving 83% inhibition at ½ MIC, followed by **C006** (81%) and **C002** (78%). **C004** exhibited comparable activity to azithromycin at ½ MIC, and all coumarin conjugates demonstrated superior activity to azithromycin at ¼ MIC and ⅛ MIC. In contrast, the tripeptides **P004** and **P007** showed remarkably weaker inhibition, particularly at ⅛ MIC (38% and 21%, respectively). Notably, these findings diverge from earlier studies, which reported that antibiofilm activity typically decreases by approximately 50% when the concentration is reduced from ½ MIC to ¼ MIC [9,17]. The superior performance of **C004** suggests a more sustained antibiofilm effect, potentially attributable to enhanced binding interactions with the LasR protein. The retention of significant activity at ¼ MIC highlights the promise of **C004**, as it indicates the potential to achieve antibiofilm activity at doses well below the MIC, thereby reducing both selective pressure for resistance and the risk of cytotoxicity.

**Figure 11:**
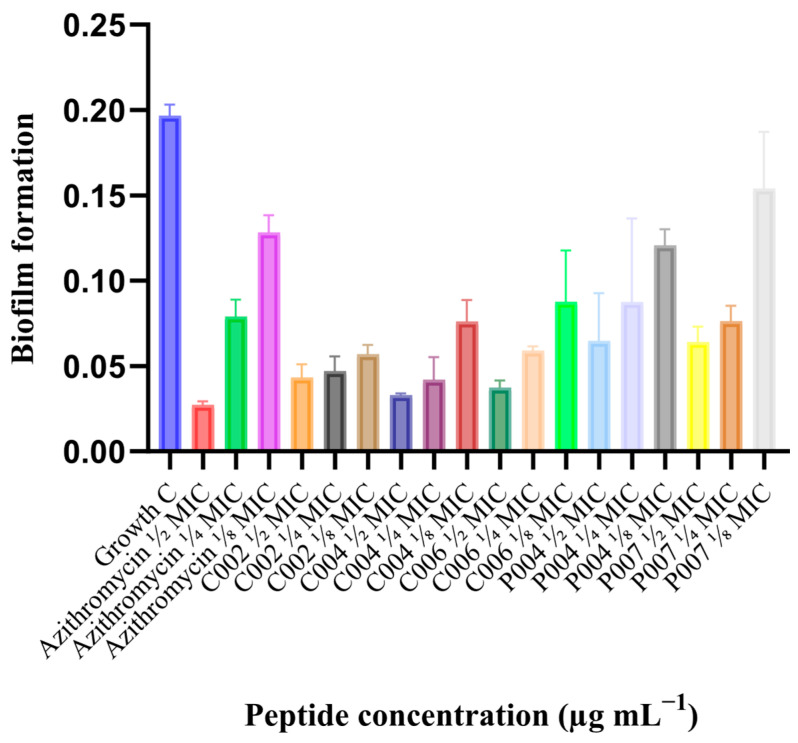
Biofilm inhibition by dipeptide coumarin conjugates (**C002**, **C004**, and **C006**), tripeptides (**P004** and **P007**), and azithromycin at ½ MIC, ¼ MIC, and ⅛ MIC. Data are presented as mean ± SD.

While the tripeptides (**P004** and **P007**) demonstrated moderate to weak antibiofilm activity, their inhibitory effects declined sharply at lower concentrations. At ⅛ MIC, **P007** achieved only 21% inhibition, consistent with the loss of activity at very low concentrations [18]. Statistical analysis using one-way ANOVA revealed significant differences among the tested peptides (F = 17.9, *p* < 0.0001), with peptide identity accounting for 88.24% of the variance (R^2^ = 0.8824), highlighting pronounced concentration- and structure-dependent effects (Figure 11). Some variability was noted among replicates, particularly at the lowest concentrations, which may reflect the intrinsic heterogeneity of biofilm formation, the sensitivity limits of the assay near detection thresholds, and minor experimental variations. Despite this variability, the reproducible inhibitory patterns across replicates confirm the robustness of the findings and reinforce the potential of these compounds as promising antibiofilm agents.

## 3. Materials and Methods

### 3.1. Ligand and Target Protein Preparation

Various peptide-based molecules have been rationally designed to target the ligand-binding domain of the LasR protein. The peptides intended to interact with the ligand-binding domain included tripeptides, dipeptides conjugated with coumarin-3-carboxylic acid, and dipeptides conjugated with dihydro-3-amino-2-(3*H*)-furanone. The names and 2D structures of these peptides are shown in Table 6 and Figure S11 (Supplementary Information). For molecular docking, which requires 3D structures with explicit hydrogen atoms, the 2D structures were converted into 3D models. MarvinSketch was used to create the 2D structures, and the 3D models were generated through MD simulations and energy minimization using the MMFF94 forcefield (Marvin version 24.3.0) ChemAxon (https://chemaxon.com/products/marvin, accessed on 14 July 2024) [66]. The resulting peptide structures were saved as PDB files. The LasR protein-autoinducer complex was retrieved from the PDB website: https://www.rcsb.org (access code 2UV0, accessed on 12 April 2024) [67]. Using UCSF Chimera, the ligand, water molecules, and chains E, F, and G were removed from the tetrameric protein structure, retaining only chain H [68]. The final purified protein structure was saved as a PDB file for subsequent molecular docking studies.

### 3.2. Molecular Docking Studies

The LasR protein was docked with each of the rationally designed molecules to predict their binding free energy (ΔG) using the Assisted Molecular Docking (AMDock) software (version 1.0), which combines the functionalities of AutoDock Vina and AutoDock4. AMDock incorporates multiple external tools, such as Open Babel (version 3.1.1, accessed on 5 September 2024), PDB2PQR, AutoLigand, and ADT scripts for structure preparation and accurate definition of the docking search space [69]. Docking poses were generated using the Lamarckian genetic algorithm, which integrates an empirical free energy function to predict both bound conformations and their associated free energies. For the target protein, polar hydrogens were assigned, and Kollman charges were applied. Regarding the ligands, Gasteiger partial charges were assigned, and non-polar hydrogen atoms were added. The ligand torsion angles were allowed full flexibility during simulations to explore conformational space comprehensively [36,70]. The grid parameters for the docking search space were defined with a centre at X: 30.10, Y: 37.10, and Z: 38.20, and dimensions of X: 30, Y: 17, and Z: 31 Å. The docking results were assessed based on binding energy values, with the conformation exhibiting the most negative ΔG selected for further analysis. This approach ensured reliable predictions of ligand-protein interactions and provided insights into the binding affinities of the ligand molecules.

### 3.3. MD Simulations of the Selected Ligands with LasR Protein

The MD simulations were carried out for the ligand-protein complexes that exhibited the most negative docking scores. Input files for the simulations were prepared using the CHARMM-GUI solution builder, with parameterization conducted via the AMBER suite version 24 and tleap tool [71]. The biomolecular components were parameterized using the AMBER force field 19SB to describe the protein dynamics accurately [72]. For the ligands, topologies were generated using the CHARMM General Force Field (CGenFF) via the CGenFF server [73]. Each MD simulation was run for a total of 100 nanoseconds (ns), and snapshots of the system were captured at intervals of 2 picoseconds (ps). This approach allowed for the detailed observation and analysis of ligand–protein interaction stability, conformational changes, and overall system dynamics over time. The protein-ligand complex systems were immersed in an octahedral TIP3P water box, extended by 1 nm in all dimensions to ensure adequate solvation [74]. Potassium and chloride ions were added to neutralize the system’s net charge [75]. Energy minimization was performed using 5000 cycles of the steepest descent algorithm to optimize the initial configurations [36]. Periodic Boundary Conditions (PBC) were applied in all directions (*x*, *y*, and *z*) [76].

System equilibration involved both NVT (constant number of particles, volume, and temperature) and NPT (constant number of particles, pressure, and temperature) ensembles. The systems were stabilized at a temperature of 300.15 K and equilibrated for 125 ps. Positional restraints of 400 kJ mol^−1^ nm^−2^ were applied to the backbone, and 40 kJ mol^−1^ nm^−2^ restraints were applied to side chains during this phase. Production runs were conducted for 100 ns in the NPT ensemble at 300.15 K and 1 bar pressure. The temperature was maintained using the Nose–Hoover thermostat, while the Parrinello–Rahman barostat controlled the pressure. The LINCS algorithm was employed to constrain hydrogen bonds, as defined in the CHARMM-GUI setup. For additional stability, the V-rescale thermostat with a coupling constant of 1 ps was employed [77,78,79,80]. Nonbonded interactions were managed using a Verlet list approach, with a nonbonded cutoff of 12 Å [81]. Long-range electrostatic interactions were computed using the Particle Mesh Ewald (PME) method [82,83].

### 3.4. MM-PBSA Calculations and Post Simulation Analysis

Molecular Mechanics-Poisson Boltzmann Surface Area (MM-PBSA) calculations were performed using g_mmpbsa (version 2.0, accessed on 12 February 2025), a GROMACS-based tool used to predict the binding affinities of the ligand-protein complexes [84]. The binary input file (.tpr) essential for this calculation was regenerated with GROMACS 5.1.4 by incorporating the molecular structure file (.gro), topology file (.top), and MD parameter file (.mdp) [85]. Key analyses of MD simulation trajectories were carried out using GROMACS utilities. The Root Mean Square Deviation (RMSD) of the ligand and protein atomic positions was calculated by aligning the protein backbone atoms via the gmx_rms subprogram. Similarly, Root Mean Square Fluctuations (RMSF) were determined for the protein’s C-alpha atoms using gmx_rmsf to assess flexibility. The radius of gyration, reflecting the compactness of the protein structure was computed using gmx_gyrate. The number of hydrogen bonds formed at the protein-ligand interface was quantified using gmx_hbond, providing insight into interaction stability. Furthermore, the gmx_distance utility was employed to calculate the center-of-mass distance between the protein and ligand throughout the simulation, offering a measure of the spatial relationship and stability of the complex [86].

### 3.5. Visualization and Plotting Software

Multiple computational tools were applied to visualize and analyze protein-ligand interactions. University of California San Francisco (UCSF) Chimera 1.16 [68] and the PyMOL Molecular Graphics System [87] were employed to examine interactions between the designed peptides and the LasR protein, measure interatomic distances, and observe conformational changes in the protein-ligand complexes. Protein–ligand interactions, including hydrophobic interactions and hydrogen bonds, were analyzed using Maestro software (version 14.0.136, 2024) [88]. The software was used to generate and visualize 2D representations of these interactions. Moreover, the trajectories visualization and analysis were performed using the Visual Molecular Dynamics (VMD) software from the Theoretical and Computational Biophysics Group (version 1.9.4a53, accessed on 28 March 2025), NIH Resource for Macromolecular Modelling and Bioinformatics at the University of Illinois at Urbana-Champaign [89].

### 3.6. ADMET Calculations

The physicochemical and pharmacokinetic properties of the designed molecules were predicted using ADMETLab 3.0 software, a computational platform widely used in the early stages of drug discovery and development to assess the absorption, distribution, metabolism, excretion, and toxicity (ADMET) profiles of potential drug candidates. The molecular structures were first converted into SMILES format using MarvinSketch 24.3.0 and subsequently uploaded to the ADMETLab 3.0 web server for property prediction (available at https://admetlab3.scbdd.com/server/screening, accessed on 3 August 2024) [90]. In addition to predicting the ADMET profiles of the selected ligands, the calculated LogP (ClogP)—a key descriptor of lipophilicity was determined for all 30 designed molecules and the autoinducer using the Manifold platform (accessible at https://app.postera.ai/mannifold, accessed on 3 August 2024).

### 3.7. Synthesis Chemistry

**Scheme 1:**
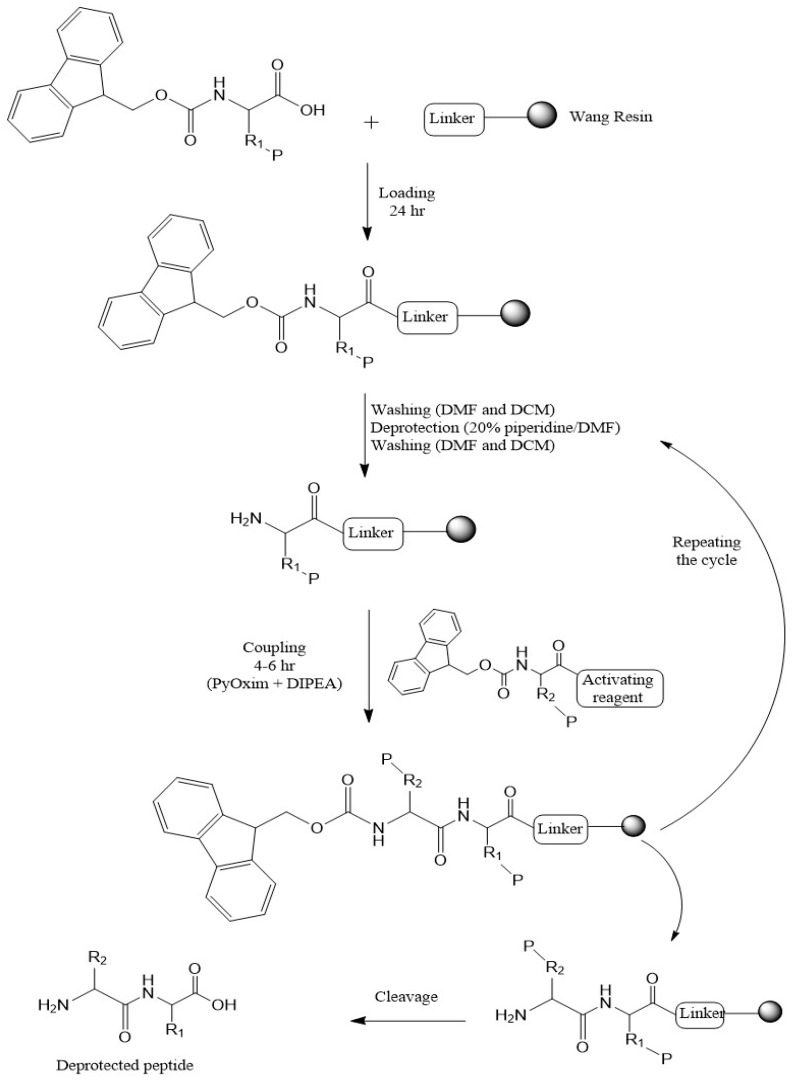
General procedure for SPPS. Coumarin conjugates were obtained using the same protocol, with coumarin-3-carboxylic acid substituting for the third amino acid and its carboxyl group activated by PyOxim and DIPEA.

The solvents and reagents used in this experiment were Peptipure^®^ grade purchased from Carl ROTH (Karlsruhe, Germany). The reactors used for the peptide synthesis were disposable poly propylene reactors also from Carl ROTH. Fmoc protected amino acids, and the resins were from Sigma-Aldrich (Darmstadt, Germany) and Merck (Darmstadt, Germany). The tripeptides (**P004** and **P007**) and dipeptide–coumarin conjugates (**C002, C004**, and **C006**) were synthesized using Fmoc-based solid-phase peptide synthesis (SPPS) on Wang resin (loading capacity: 0.7 mmol/g) [91,92]. Fmoc deprotection was performed using 20% piperidine in DMF (2 × 10 min). Coupling reactions were carried out using PyOxim (4 equiv.) and DIPEA (8 equiv.) in DMF as the solvent. Coupling completion was monitored by Kieser test (modified ninhydrin test) for primary amines and p-chloranil test for secondary amine (proline). After completion of chain assembly, peptides were cleaved from the resin using a trifluoroacetic acid (TFA)-based cleavage cocktail [93,94]. Following cleavage, TFA was removed under reduced pressure using a rotary evaporator, the peptides were dissolved in mixture of acetonitrile: water 1:1 then freeze-dried (Scheme 1). The peptides were analyzed by ^1^H NMR and electrospray ionization mass spectrometry (ESI-MS) to confirm structure and purity. Isolated yields ranged from 60% to 80%.

### 3.8. Antibacterial Activities

#### 3.8.1. Determination of Minimum Inhibitory Concentration

The minimum inhibitory concentrations (MICs) of the synthesized compounds were evaluated against *P. aeruginosa* (ATCC 27853) using the broth microdilution method, following the Clinical and Laboratory Standards Institute (CLSI) guidelines with minor modifications [16,24]. A single bacterial colony was cultured on cetrimide agar under aerobic conditions at 37 °C for 24 h. 100 µL of Mueller Hinton broth (MH Broth) was dispensed into each well of a 96-well flat-bottom polypropylene microtiter plate. Two-fold serial dilutions of the synthetic peptides were prepared in the wells, with final concentrations ranging from 2048–32 µg/mL. A bacterial suspension, obtained from an overnight culture and adjusted to an optical density of OD_600_ = 0.1 (approximately 0.5 McFarland standard), was further diluted to achieve a final inoculum of 5 × 10^5^ CFU/mL in each well. Wells containing 200 µL of sterile medium used as sterility controls, while those containing 200 µL of bacterial suspension without peptides used as growth controls. The plates were incubated overnight at 37 °C, and bacterial growth was measured by reading the optical density at 600 nm using microplate reader. All experiments were conducted in triplicate [16,95].

#### 3.8.2. Antibiofilm Activity Assay

The antibiofilm activity of the synthesized compounds was assessed using the crystal violet staining method in 96-well flat-bottom polypropylene microtiter plates. A single colony of *P. aeruginosa* was inoculated into tryptic soy broth (TSB) and incubated at 37 °C for 24 h. The resulting culture was diluted to approximately 5 × 10^5^ CFU/mL and dispensed into the wells containing the test compounds, yielding a final volume of 200 µL per well. The plates were incubated at 37 °C for 24 to 48 h to facilitate biofilm formation. Following incubation, wells were gently washed with phosphate-buffered saline (PBS) to remove non-adherent (planktonic) cells. Biofilms were fixed by air drying at room temperature and subsequently stained with 0.1% crystal violet for 15 min. Excess stain was removed by washing with distilled water, and the bound dye was solubilized using an ethanol:acetone mixture (80:20, *v*/*v*). Biofilm biomass was quantified by measuring absorbance at 595 nm (OD_595_). The percentage of biofilm inhibition was determined at sub-MIC and two lower concentrations of the tested compounds, relative to untreated controls (without peptides) as the 100% biofilm control. All assays were performed in triplicate [24,38,96]. The data are presented as mean ± SD. All statistical analyses were performed using GraphPad Prism version 10.5.0. The difference between different treatment groups was determined by using a one-way ANOVA.

## 4. Conclusions

This work successfully combined computational and experimental approaches to develop peptide-based inhibitors targeting QS-mediated biofilm formation in *P. aeruginosa*. Among the synthesized compounds, coumarin conjugates, particularly **C004**, showed activity comparable to that of azithromycin at ½ MIC, whereas it outperformed azithromycin at ¼ MIC and ⅛ MIC. **C004** showed the strongest antibiofilm activity, aligning with their predicted high binding affinity and favorable ADMET properties. While the MIC values were moderate, the sustained inhibition of biofilm formation at sub-MIC levels underscores the therapeutic potential of these compounds as anti-virulence agents. Future work should focus on optimizing physicochemical properties, improving antibacterial potency, and validating efficacy in relevant in vivo infection models. Collectively, these findings support the development of peptide–coumarin conjugates as novel strategies to combat biofilm-associated infections and antimicrobial resistance.

## Figures and Tables

**Table 1:** Molecular docking results, ClogP, and MWs for the autoinducer and the designed peptide-based inhibitors.

Code	∆G (kcal/mol)	MW (g/mol)	ClogP
OdDHL	−8.45	297.39	2.95
**P001**	−4.92	328.37	−1.96
**P002**	−4.52	341.45	0.97
**P003**	−5.25	283.32	−0.68
**P004**	−9.19	300.36	−1.45
**P005**	−7.98	349.38	−0.16
**P006**	−5.69	394.43	−1.79
**P007**	−8.12	366.41	−0.92
**P008**	−6.25	335.36	−0.55
**C001**	−5.62	375.38	0.22
**C002**	−9.18	360.36	1.14
**C003**	−7.13	389.40	0.61
**C004**	−10.35	374.39	1.53
**C005**	−8.97	360.36	1.14
**C006**	−9.73	344.32	0.6
**C007**	−8.4	417.42	−0.26
**C008**	−4.61	403.39	−0.64
**C009**	−7.73	348.31	−0.53
**C010**	−7.25	334.28	−0.92
**C011**	−7.89	360.36	1.14
**F001**	−7.43	314.34	−2.88
**F002**	−8.24	328.37	−2.49
**F003**	−8.81	286.33	−2.01
**F004**	−9.01	271.31	−1.09
**F005**	−9.08	271.31	−1.09
**F006**	−8.53	300.35	−1.62
**F007**	−9.49	286.33	−2.01
**F008**	−9.19	255.27	−1.71
**F009**	−8.22	259.26	−2.76
**F010**	−7.31	245.23	−3.15
**F011**	−8.37	271.31	−1.09

**Table 2:** Binding free energy results of MM-PBSA for the selected compounds.

Code	Sequence	MM-PBSA (kcal/mol)
**C002**	IG-coumarin	−35.96
**C004**	LA-coumarin	−50.10
**C006**	PG-coumarin	−45.55
**F007**	KG-furanone	−43.27
**F008**	PG-furanone	−34.29
**P004**	PGK	−55.45
**P007**	YGK	−45.73

**Table 3:** Prediction of pharmacokinetic results of the selected compounds.

Comp.	Absorption ^a^ HIA	Distribution		Metabolism				Excretion	
		^b^ PPB (%)	^c^ BBB	CYP2D6-inh	CYP2D6-sub	CYP3A4-inh	CYP3A4-sub	CL-Plasma	t_0.5_
**C002**	0.00	0.00	0.00	0.00	0.00	0.00	0.00	0.00	0.00
**C004**	0.02	76.80	0.00	0.00	0.00	0.00	0.00	2.07	1.06
**C006**	0.00	80.14	0.00	0.00	0.05	0.00	0.00	2.29	0.87
**F007**	0.42	8.63	0.00	0.00	0.24	0.00	0.03	4.20	1.02
**F008**	0.33	11.57	0.02	0.00	0.03	0.01	0.00	3.83	1.56
**P004**	0.88	8.47	0.05	0.00	0.00	0.00	0.00	3.11	1.38
**P007**	0.04	12.59	0.00	0.00	0.87	0.00	0.00	5.19	0.96

^a^ HIA: human intestinal absorption (empirical decision: 0–0.3: excellent; 0.3–0.7: medium; 0.7–1.0: poor); ^b^ PPB: plasma protein binding, optimal ≤ 90%; ^c^ BBB: blood–brain barrier 0–0.1 (excellent), 0.1–0.3 (medium), 0.3–0.5 (poor); CL-plasma: unit is mL/min/kg (more than 15; high clearance, 5–15; moderate clearance, less than 5; low clearance); t_0.5_: unit is hour (less than 1 h = ultra short half-life, 1–4 h = short half-life, 4–8 h = intermediate half-life, more than 8 h = long half-life); CYP2D6 and CYP3A4 inhibitor (substrate); category 0: non-substrate/non-inhibitor; category 1: substrate/inhibitor. The output value is the probability of being substrate/inhibitor, within the range of 0–1.

**Table 4:** Prediction of Physicochemical properties, hepatotoxicity, cardiotoxicity, and carcinogenicity of the selected ligands.

Comp.	MW.	Lipinski	TPSA	logP	Toxicity		Carcinogenicity
					hERG-Blocker	H-HT	
**C002**	360.13	0.00	125.71	1.47	0.03	0.26	0.34
**C004**	374.15	0.00	125.71	2.57	0.02	0.43	0.25
**C006**	344.1	0.00	116.92	0.86	0.05	0.71	0.54
**F007**	286.16	0.00	136.54	−2.67	0.19	0.48	0.40
**F008**	255.12	0.00	96.53	−2.07	0.18	0.43	0.24
**P004**	300.36	0.00	133.55	−2.84	0.15	0.48	0.07
**P007**	366.19	0.00	167.77	−2.02	0.17	0.69	0.02

TPSA: topological polar surface area, optimal 0–140; Lipinski: 0 passed; H-HT: human hepatotoxicity: category 1: H-HT positive, category 0: H-HT negative; the output value 0–1 is the probability of being toxic; hERG-blocker: category 1 is hERG+, category 0 is hERG−, the output value 0–1 is the probability of being hERG+; carcinogenicity: category 1 is carcinogens, category 0 is non carcinogens, the output value 0–1 is the probability of being toxic.

**Table 5:** MIC values for the synthesized compounds and azithromycin as the positive control.

Code	MIC (μg/mL)
**P004**	1024
**P007**	1024
**C002**	512
**C004**	512
**C006**	512
Azithromycin	256

**Table 6:** The designed tripeptide, dipeptide–coumarin conjugates, and dipeptide–furanone conjugates.

Tripeptides	
**P001**	RGP
**P002**	LPL
**P003**	PAP
**P004**	PGK
**P005**	YAP
**P006**	YGR
**P007**	YGK
**P008**	YGP
**Coumarin-3-carboxylic Acid Conjugates** 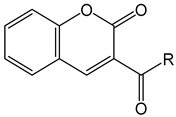	
**C001**	R = GK
**C002**	R = IG
**C003**	R = KA
**C004**	R = LA
**C005**	R = LG
**C006**	R = PG
**C007**	R = RA
**C008**	R = RG
**C009**	R = SA
**C010**	R = SG
**C011**	R = VA
**3-Aminodihydrofuran-2(3*H*)-one Conjugates** 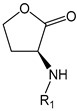	
**F001**	R1 = RG
**F002**	R1 = RA
**F003**	R1 = GK
**F004**	R1 = IG
**F005**	R1 = LG
**F006**	R1 = KA
**F007**	R1 = KG
**F008**	R1 = PG
**F009**	R1 = SA
**F010**	R1 = SG
**F011**	R1 = VA

## Data Availability

Data presented in this study are contained within the article or Supplementary Material.

## Supplementary Materials

The following supporting information can be downloaded at: https://www.mdpi.com/article/10.3390/ph18101572/s1, Figures S1–S5: ^1^H NMR result of **C002**, **C004**, **C006**, **P004**, and **P007**; Figures S6–S10: Mass spectroscopy result of **C002**, **C004**, **C006**, **P004**, and **P007**; Figure S11: Structures of the designed tripeptide, dipeptides-coumarin conjugates, and dipeptide-furanone conjugates; Figures S12–S16: ^13^C NMR result of **C002, C004, C006, P004,** and **P007.**
