# Supplementary material for: Rational Design, Computational Analysis and Antibacterial Activities of Synthesized Peptide-Based Molecules Targeting Quorum Sensing-Dependent Biofilm Formation in Pseudomonas aeruginosa
Source: Pharmaceuticals (Basel). 2025 Oct 18;18(10):1572. doi: 10.3390/ph18101572 (PMC12567134; doi:10.3390/ph18101572)
Supplement: Supplementary file 1 [file pharmaceuticals-18-01572-s001.zip › pharmaceuticals-3913213-supplementary.pdf]

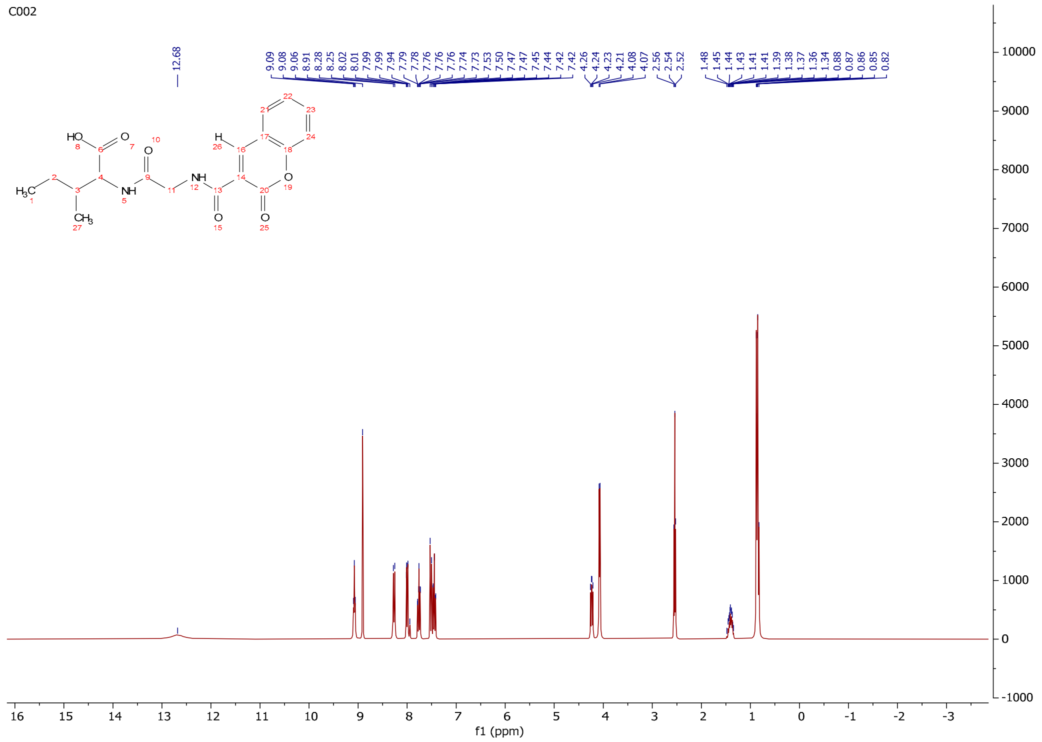

Figure S1. ^{1}HNMR result of **C002**

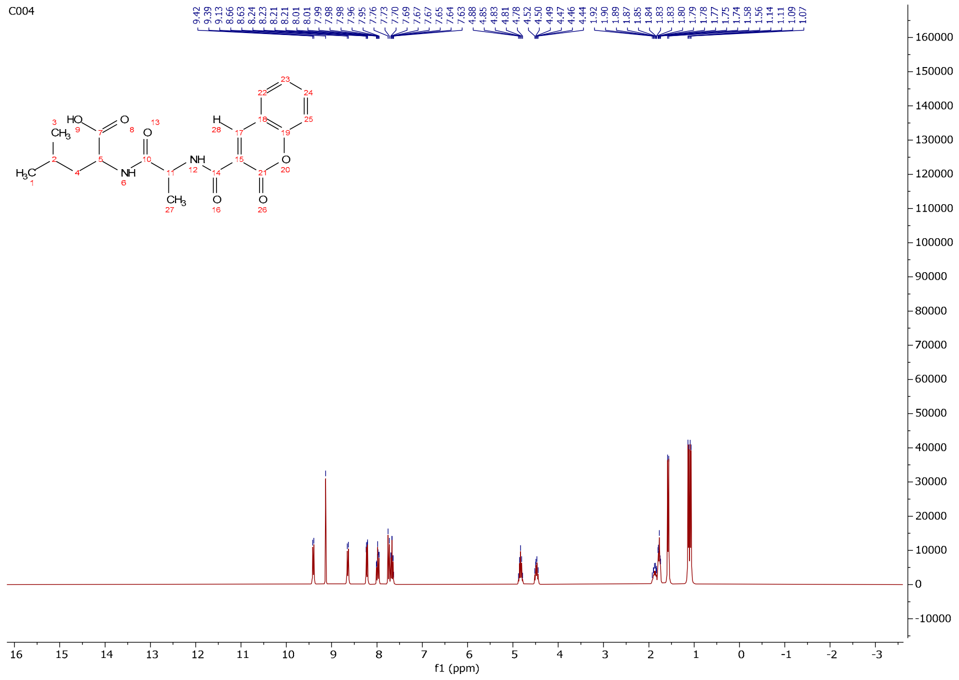

Figure S2. ^{1}HNMR result of **C004**

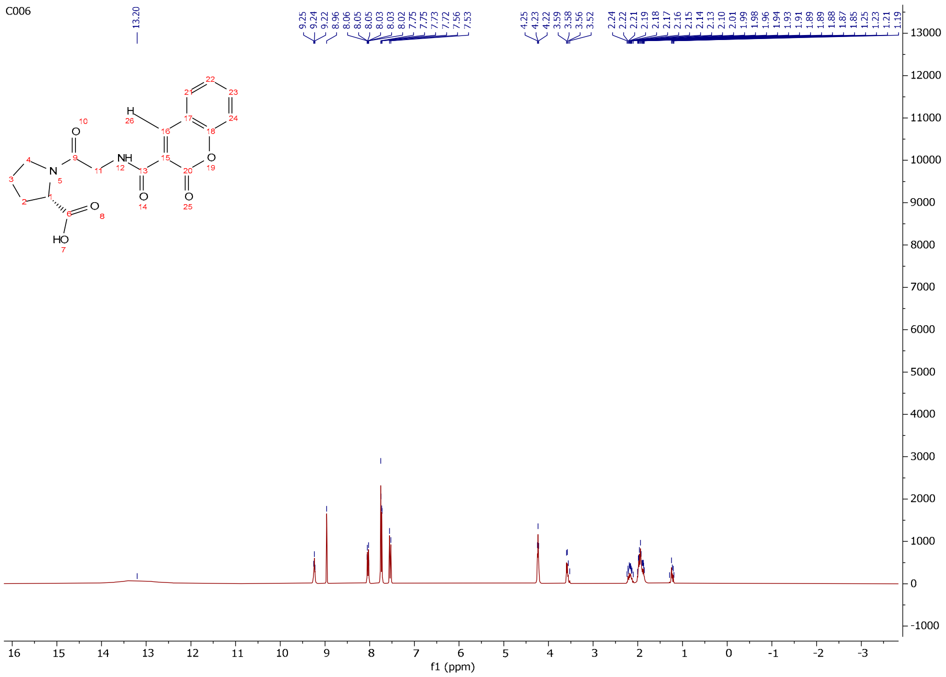

Figure S3. ^{1}HNMR result of **C004**

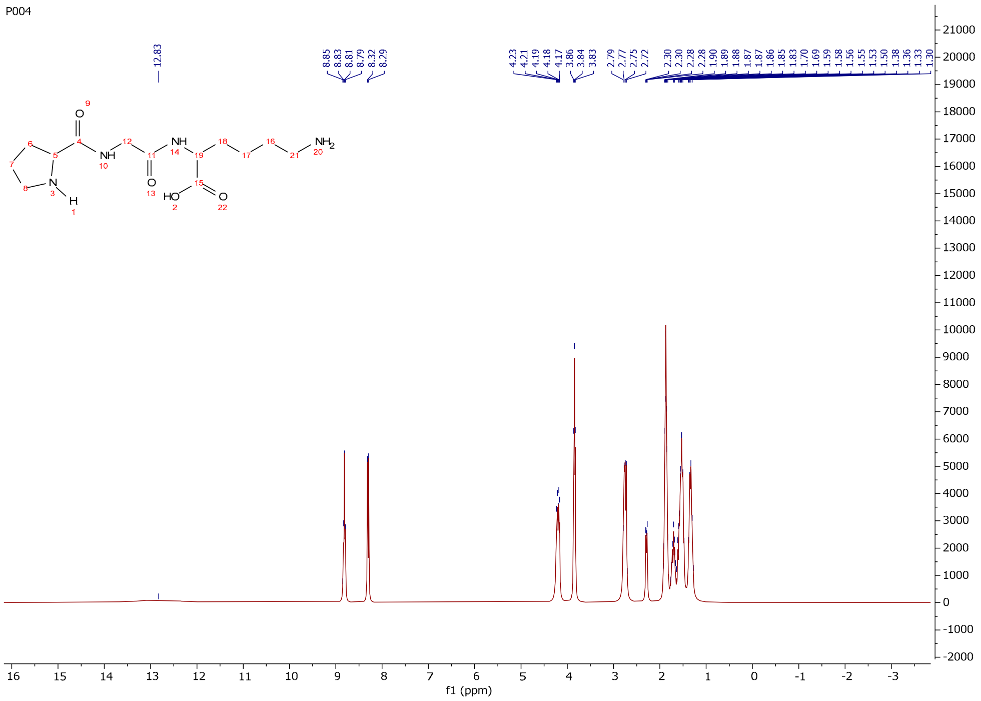

Figure S4. ^{1}HNMR result of **P004**

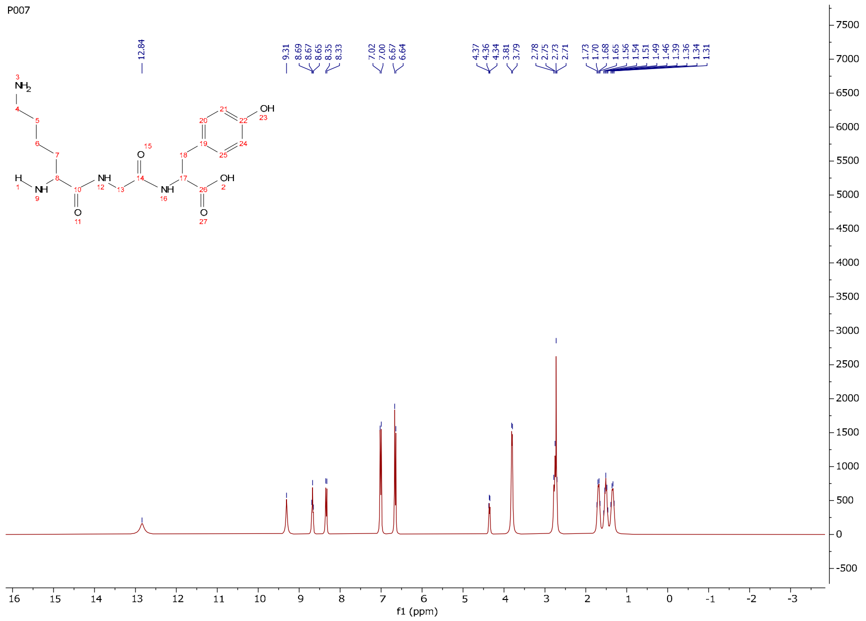

Figure S5. ^{1}HNMR result of **P007**

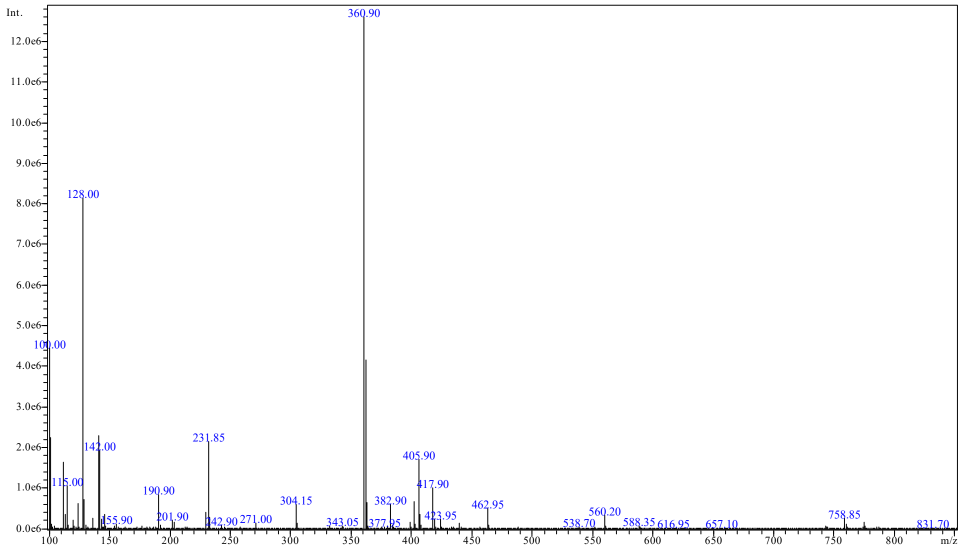

Figure S6. Mass spectroscopy result of **C002**

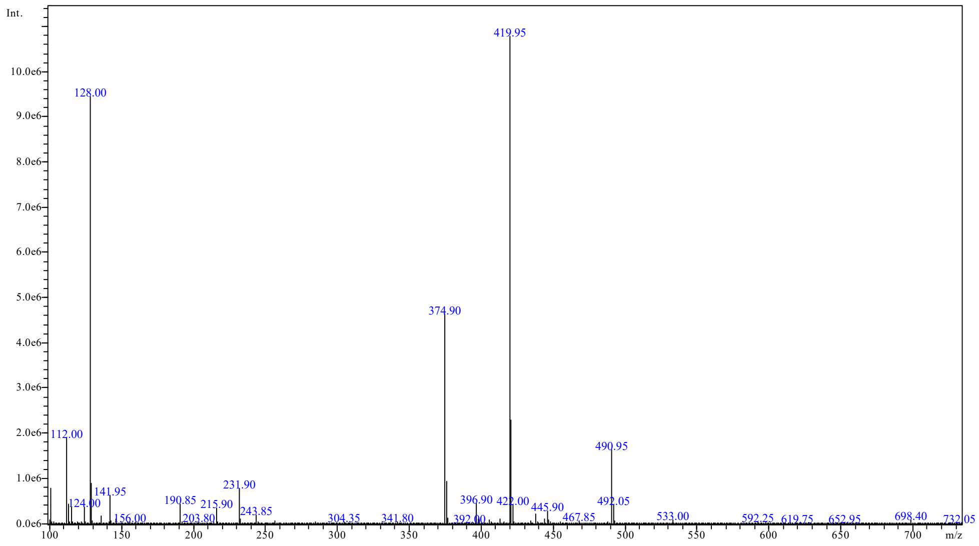

Figure S7. Mass spectroscopy result of **C004**

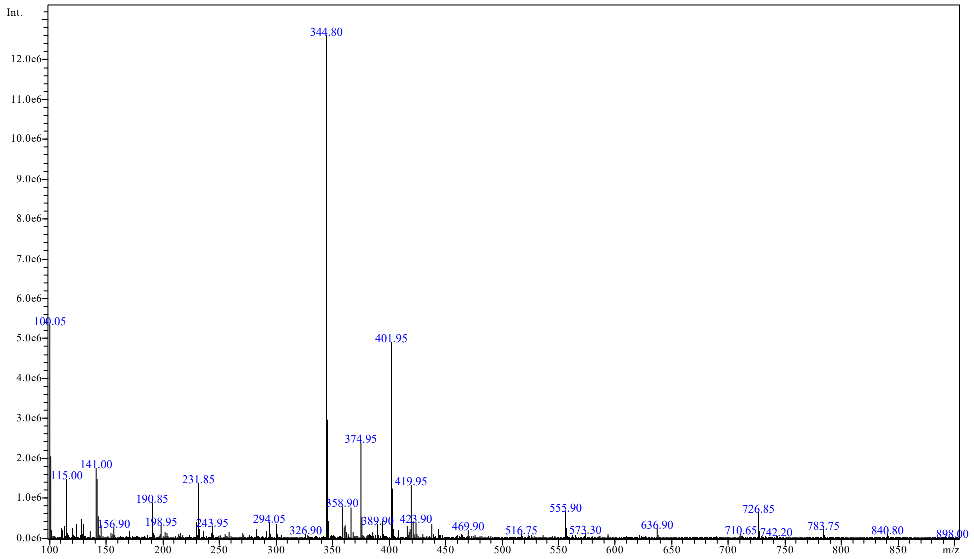

Figure S8. Mass spectroscopy result of **C006**

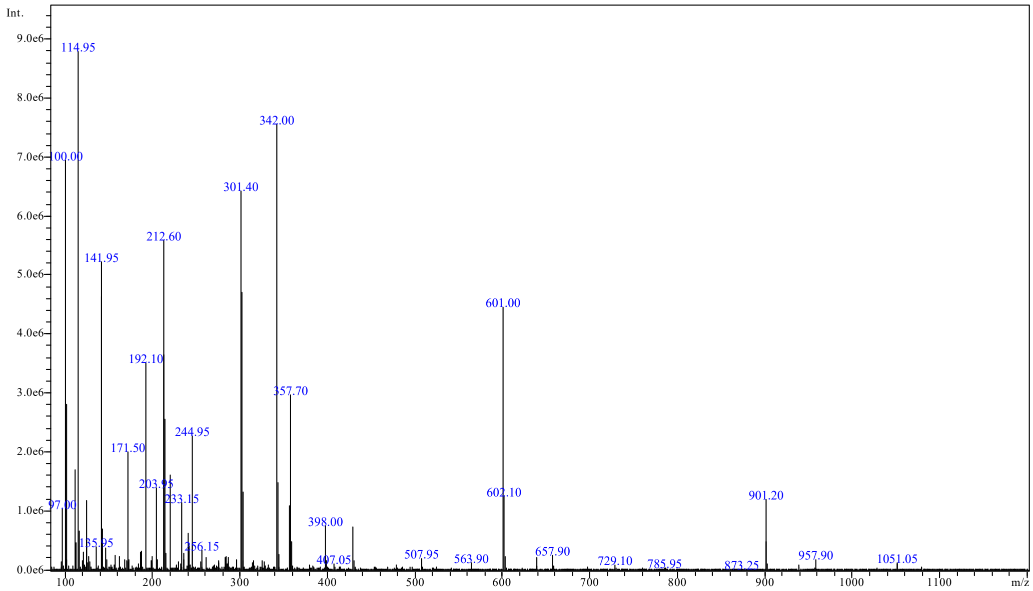

Figure S9. Mass spectroscopy result of **P004**

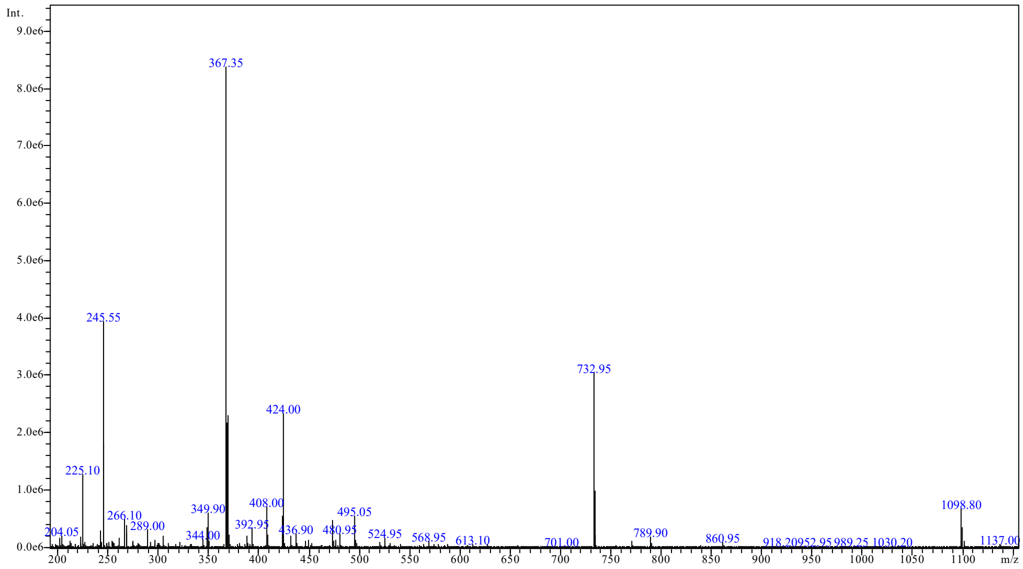

Figure S10. Mass spectroscopy result of **P007**

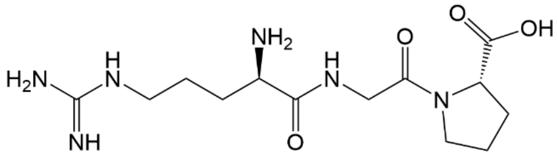

P001

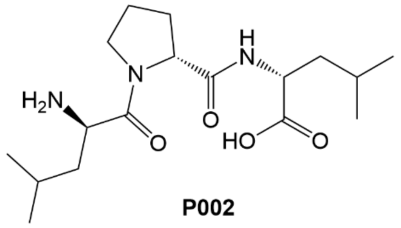

P002

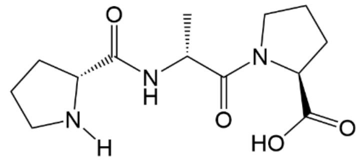

P003

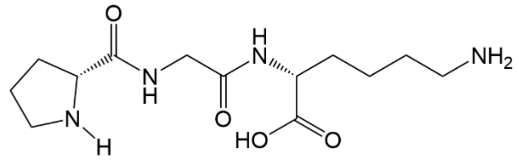

P004

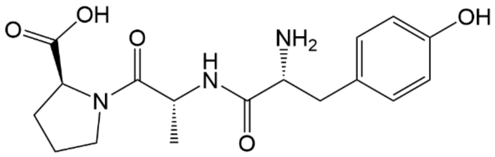

P005

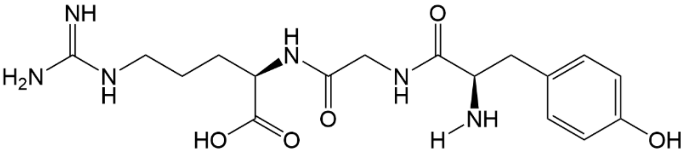

P006

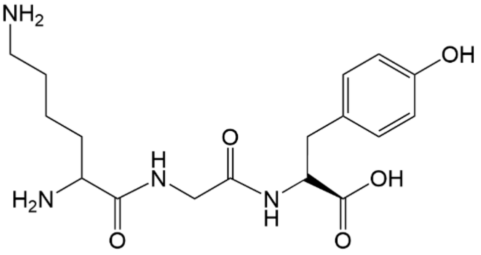

P007

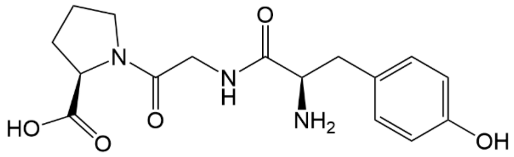

P008

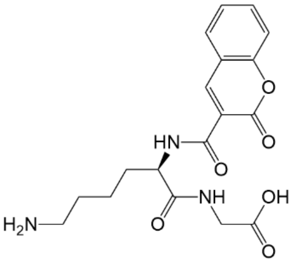

C001

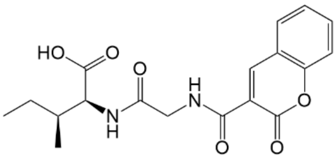

C002

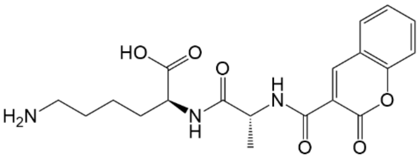

C003

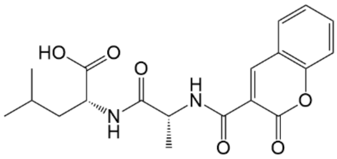

C004

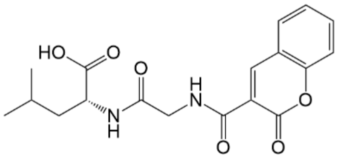

C005

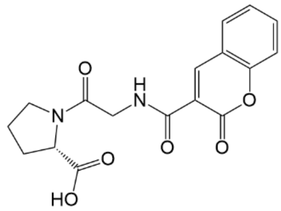

C006

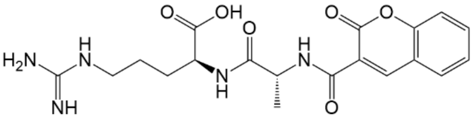

C007

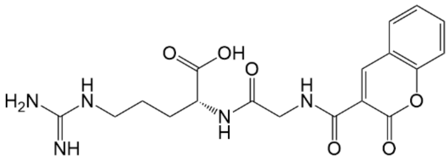

C008

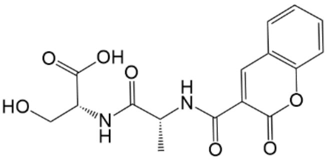

C009

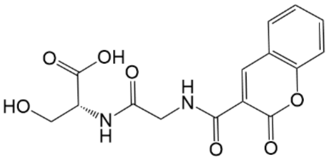

C010

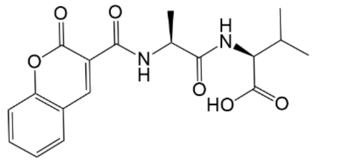

C011

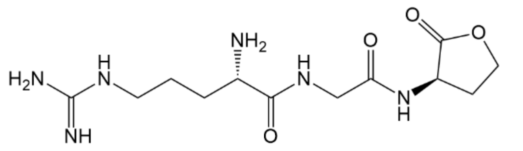

F001

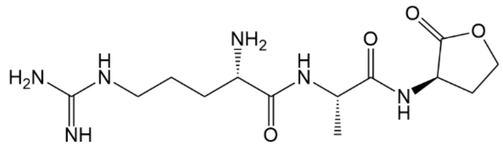

F002

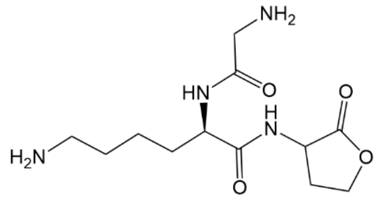

F003

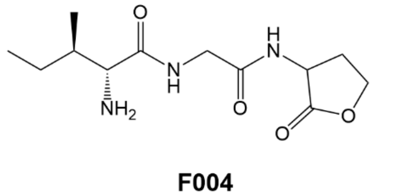

F004

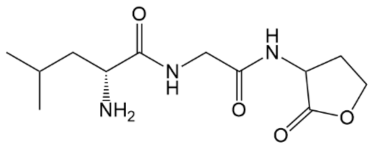

F005

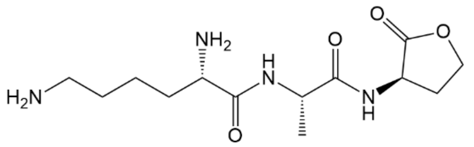

F006

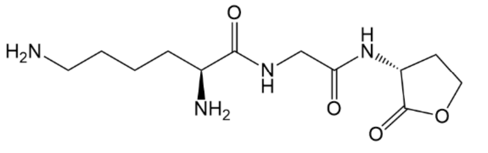

F007

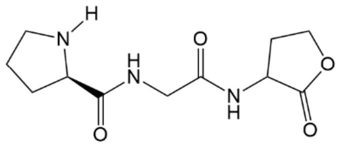

F008

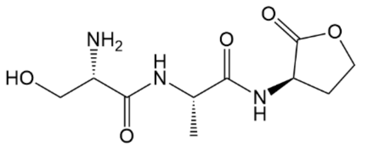

F009

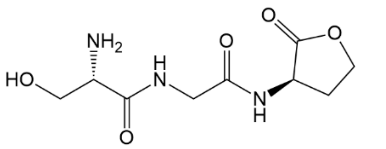

F010

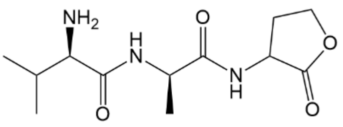

F011

Figure S11. Structures of the designed tripeptide, dipeptides-coumarin conjugates, and dipeptide-furanone conjugates.

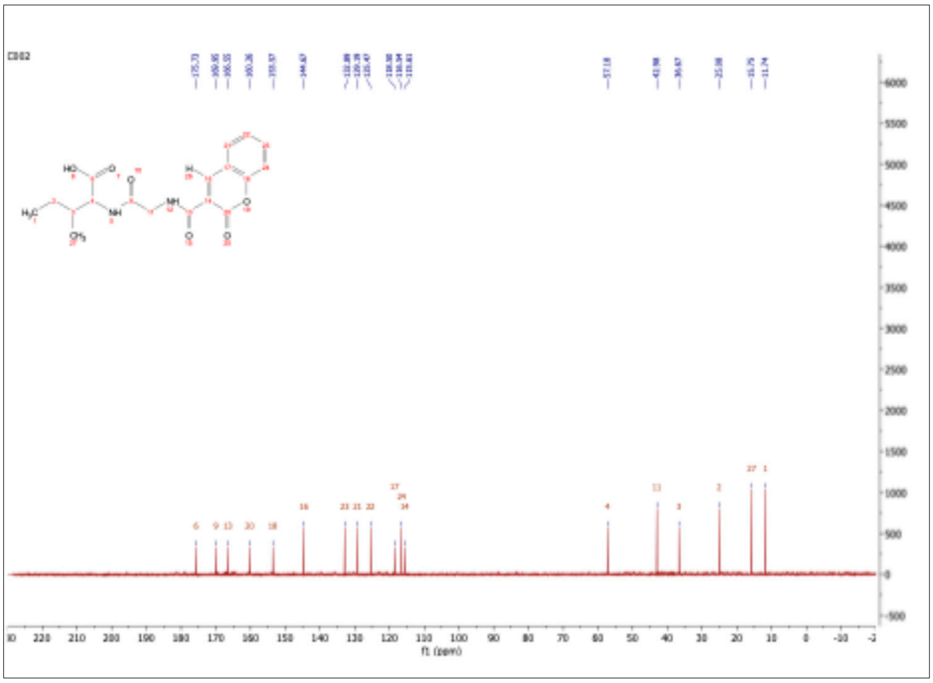

Figure S12. ^{13}CNMR result of **C002**.

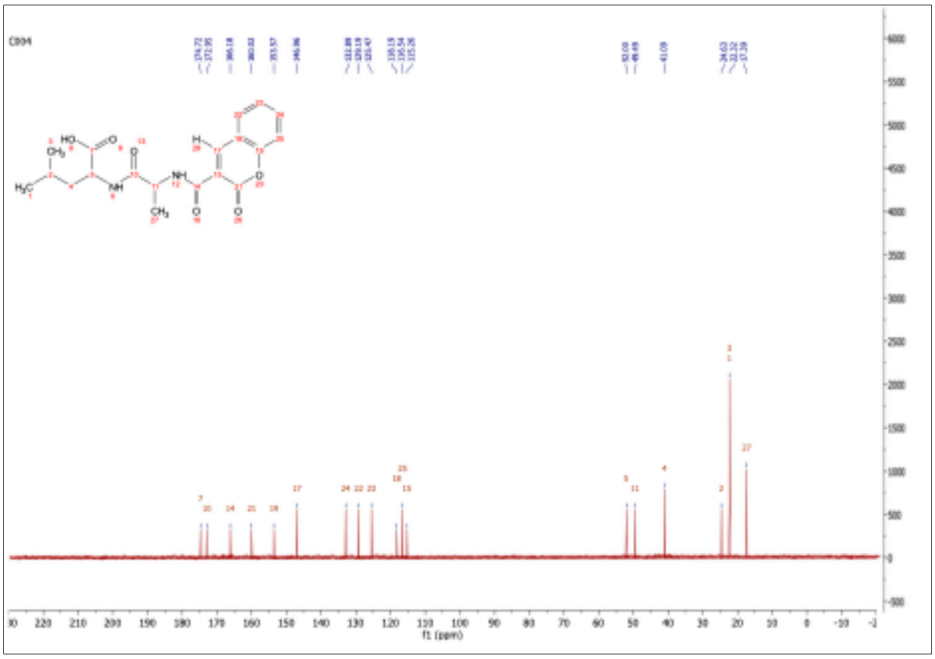

Figure S13. ^{13}CNMR result of **C004**.

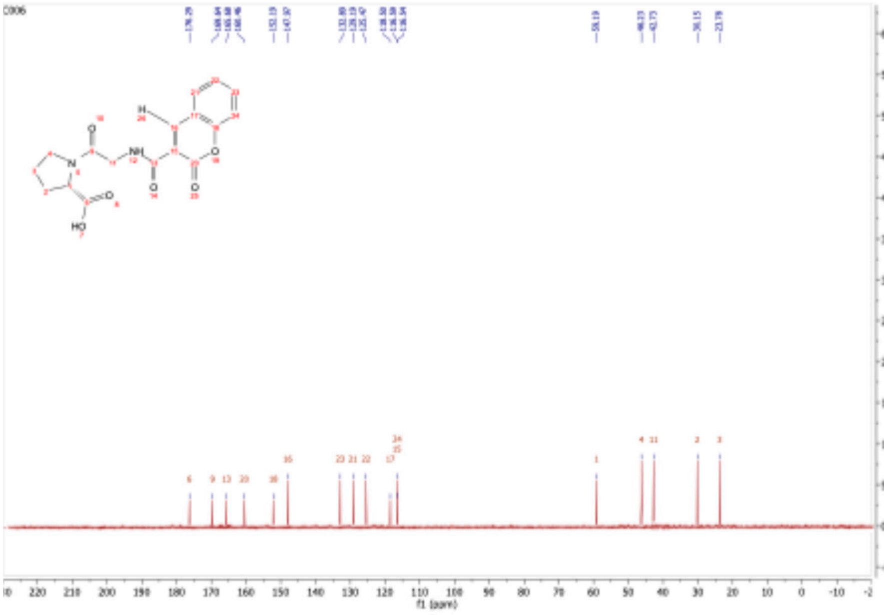

Figure S14. ^{13}CNMR result of **C006**.

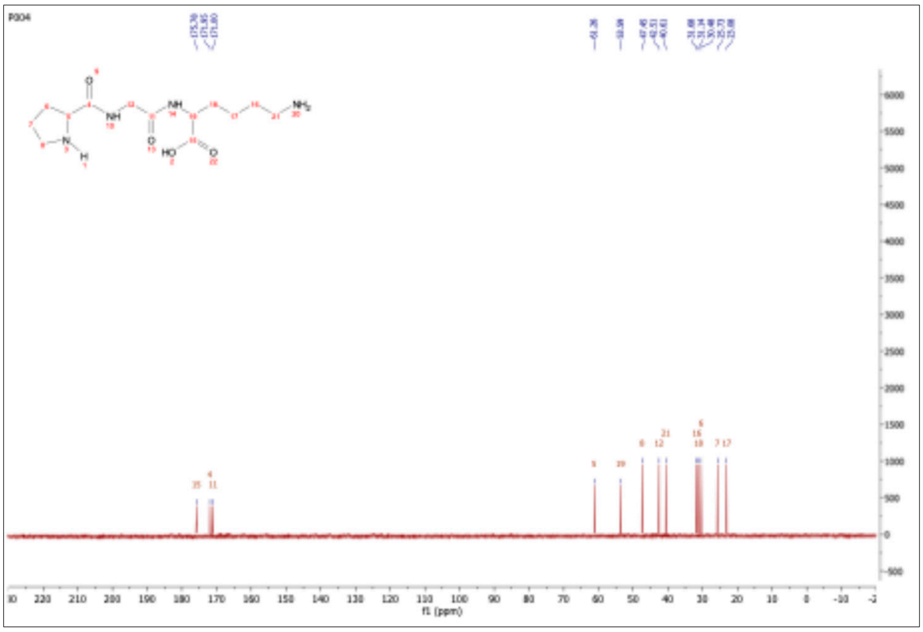

Figure S15. ^{13}CNMR result of **P004**.

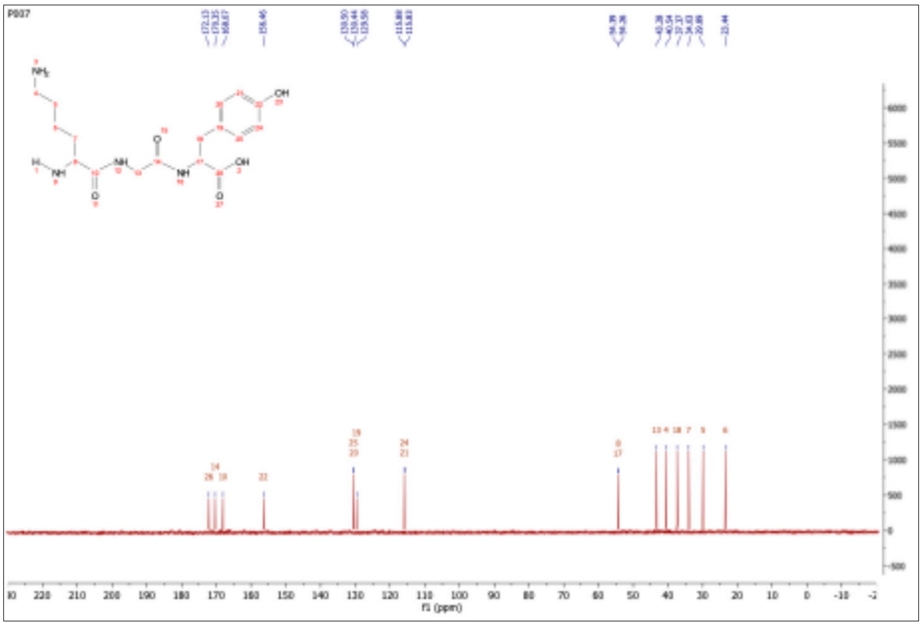

Figure S16. ^{13}CNMR result of **P007**.
